# Identification of PANoptosis-Based Prognostic Signature for Predicting Efficacy of Immunotherapy and Chemotherapy in Hepatocellular Carcinoma

**DOI:** 10.1155/2023/6879022

**Published:** 2023-06-05

**Authors:** Xiaofeng Xiong, Qianben Song, Mengjia Jing, Wei Yan

**Affiliations:** Department of Gastroenterology, Tongji Hospital, Tongji Medical College, Huazhong University of Science and Technology, Wuhan, China

## Abstract

**Background:**

PANoptosis has been a research hotspot, but the role of PANoptosis in hepatocellular carcinoma (HCC) remains widely unknown. Drug resistance and low response rate are the main limitations of chemotherapy and immunotherapy in HCC. Thus, construction of a prognostic signature to predict prognosis and recognize ideal patients for corresponding chemotherapy and immunotherapy is necessary.

**Method:**

The mRNA expression data of HCC patients was collected from TCGA database. Through LASSO and Cox regression, we developed a prognostic signature based on PANoptosis-related genes. KM analysis and ROC curve were implemented to evaluate the prognostic efficacy of this signature, and ICGC and GEO database were used as external validation cohorts. The immune cell infiltration, immune status, and IC50 of chemotherapeutic drugs were compared among different risk subgroups. The relationships between the signature and the efficacy of ICI therapy, sorafenib treatment, and transcatheter arterial chemoembolization (TACE) therapy were investigated.

**Result:**

A 3-gene prognostic signature was constructed which divided the patients into low- and high-risk subgroups. Low-risk patients had better prognosis, and the risk score was proved to be an independent predictor of overall survival (OS), which had a well predictive effect. Patients in high-risk population had more immunosuppressive cells (Tregs, M0 macrophages, and MDSCs), higher TIDE score and TP53 mutation rate, and elevated activity of base excision repair (BER) pathways. Patients with low risk benefited more from ICI, TACE, and sorafenib therapy. The predictive value of the risk score was comparable with TIDE and MSI for OS under ICI therapy. The risk score could be a biomarker to predict the response to ICI, TACE, and sorafenib therapy.

**Conclusion:**

The novel signature based on PANoptosis is a promising biomarker to distinguish the prognosis predict the benefit of ICI, TACE, and sorafenib therapy, and forecast the response to them.

## 1. Introduction

Hepatocellular carcinoma (HCC), the most common type of liver cancer with poor prognosis, ranks as the third highest cause of cancer-related death currently around the world with nearly 900 thousand new cases in 2020 [1]. According to the prediction of the World Health Organization, about 1 million HCC patients will die of HCC in 2030 [2]. Although surgical treatment based on radical surgery has greatly improved the prognosis of patients, the five-year survival rate of HCC is only 12% [3]. Sorafenib and other clinical first-line drugs have a certain therapeutic effect on advanced HCC, but drug resistance is increasingly common. In recent years, immune checkpoint inhibitor (ICI) therapy for HCC patients is a burgeoning field of study, which has attracted a great deal of attention and also achieved promising results [4]. However, the low response rate to ICI therapy is a major deficiency which is still unresolved, and there were fewer biomarkers to predict prognosis and response to ICI therapy. Therefore, identification of a potential predictor for the efficacy of ICI therapy is necessary for individualized immunotherapy in HCC.

Due to the broad crosstalk among programmed cell death (PCD) pathways, PCD generally occur not alone but in a mixed mode, just like pyroptosis, apoptosis, and necroptosis [5]. To better apprehend the crosstalk among them, PANoptosis is defined as a type of inflammatory cell death driven by PANoptosome, which contains the key features of pyroptosis, apoptosis, and necroptosis [6]. Similar to other PCD, PANoptosis induces tumor cell death to inhibit the development of cancer, which can be a therapeutic target for oncotherapy. Moreover, there exists a close relationship between innate immune and PANoptosis, and the molecules composed of PANoptosome involve in inflammatory immune responses [7]. PANoptosis also has an important impact on the PD-1/PD-L1 pathway [8]. Many chemotherapeutic drugs can cause pyroptosis, apoptosis, or necroptosis to kill tumor cells, especially sorafenib, the clinical first-line drug for advanced HCC patients which can induce the three types of PCD concurrently [9–11]. Thus, inducing PANoptosis may decrease the drug resistance to sorafenib therapy.

Given that the close relationship between PANoptosis and innate immune, and chemotherapeutic agents, identification of a novel biomarker based on PANoptosis to select ideal HCC patients for ICI therapy and sorafenib treatment has good feasibility, which can aid in the execution of individualized treatment and improve the outcome of HCC patients. To predict the overall survival (OS) of HCC patients and explore whether the efficacy of ICI and sorafenib therapy can be forecasted by PANoptosis-related molecules, we construct a prognostic signature made up of three PANoptosis-related genes using LASSO and Cox regression. The cohorts of ICI therapy, transcatheter arterial chemoembolization (TACE), and sorafenib treatment were utilized to evaluate the predictive value of this signature for ICI, TACE, and sorafenib therapy in HCC.

## 2. Materials and Methods

### 2.1. Data Retrieval

The mRNA data and corresponding clinical information of 50 normal liver tissues and 374 HCC specimens were downloaded from TCGA database. To ensure the accuracy of our study, we performed external validation in which we retrieved the transcript and clinical information of 243 HCC specimens from ICGC Data Portal and 221 HCC samples from the GEO database (GSE14520). 26 PANoptosis-related genes were extracted from the previous articles which were shown in Supplementary Table S1 [5, 6].

### 2.2. Consensus Clustering Analysis Based on DEGs

To investigate the connections between differentially expressed PANoptosis-related genes and hepatocellular carcinoma subtypes, consensus clustering analysis was carried out with the “ConsensusClusterPlus” R package. Principal component analysis (PCA) was implemented by the “stats” R package, and K-M analysis was employed to compare the OS among different clusters. Differentially expressed genes (DEGs) among clusters were identified through the “limma” package. To explore the functional differences between the two clusters, we adopted Gene Set Enrichment Analysis (GSEA) to analyze.

### 2.3. Construction of a Prognostic Model Based on PANoptosis-Related Genes

In TCGA cohort, differentially expressed PANoptosis-related genes (DEPGs) were identified from a list of 26 PANoptosis-related genes by the “limma” R package, in which the DEGs with a false discovery rate (FDR) < 0.05 were picked out. To screen out the differentially expressed prognostic PANoptosis-related genes (DEPPGs), univariate Cox regression of OS and K-M analysis were applied. Afterwards, the DEPPGs were enrolled in LASSO Cox regression analysis using the “glmnet” R package. Multivariate Cox regression analysis was employed for the genes picked out from LASSO regression through the “survival” R package to construct a prognosis-predicting model. Based on the expression values of each gene and their regression coefficients, a risk score for each sample was calculated based on the following formula: risk score = ∑*i* Coefficient (DEPPGi) *∗* Exp (DEPPGi). Patients were classified into low- and high-risk groups on the basis of the median risk score for subsequent analysis. PCA was adopted to evaluate distinction between the two groups. Similarly, HCC samples in the validation cohorts (ICGC and GSE14520) were also divided into low- or high-risk subgroups according to the median risk score based on the same formula.

### 2.4. Analysis of Immune Cell Infiltration in Risk Subgroups

The potential association among the 3 genes in the signature and immune cell infiltration was evaluated through the TIMER2 online tool (https://timer.cistrome.org). To assess the immune infiltration among different risk subgroups, the CIBERSORTx algorithm was implemented to quantify the proportions of 22 types of immune cells. In addition, single sample gene set enrichment analysis (ssGSEA) was also implemented to explore the relationship between risk scores and immune cell infiltration using the “ESTIMATE” R package. In addition, the expression levels of 24 immune cell markers were collected and correlation analysis was utilized to investigate the relationship between risk value and immune-infiltrating cell markers.

### 2.5. Prediction of Immunotherapeutic Response

To investigate the connection between prognostic signature and immunotherapeutic response, the expression of immunotherapy-related genes was compared among risk subgroups. Moreover, the data of significant immunotherapy indicators including tumor immune dysfunction and exclusion (TIDE), microsatellite instability (MSI), and myeloid-derived suppressor cells (MDSC) were acquired online (https://tide.dfci.harvard.edu/), while another immunotherapy marker, immunophenotypic score (IPS) which represented the response to PD-1 and CTLA-4 inhibitor, was obtained from Cancer Immunome Atlas (TCIA, https://tcia.at/). Difference analysis of TIDE, MSI, MDSC, and IPS was employed in low- and high-risk population. Anti-PD-L1 cohort (GSE78220) and immune checkpoint blockade cohort (anti-PD-L1 and anti-CTLA4, GSE91061) were downloaded to verify the predictive value of risk values for immunotherapy responses.

### 2.6. Correlation of the Prognostic Signature with Chemotherapeutic Agents' Sensitivity

The half-maximal inhibitory concentration (IC50) of chemotherapeutic drugs was computed based on the “oncoPredict” R package [12]. The lower values of IC50 represented better sensitivity to chemotherapeutic agents. The sorafenib cohort (GSE109211) and transcatheter arterial chemoembolization cohort (TACE, GSE104580) were utilized to perform stratified analysis and ROC curve to estimate the predictive value of the risk score for the response to chemotherapy.

### 2.7. Comprehensive Analysis of Molecular Characteristics in Different Subgroups

To further explore the biological function of differentially expressed genes, gene ontology (GO) enrichment analysis and Kyoto Encyclopedia of Genes and Genomes (KEGG) pathway enrichment analysis were employed between the two risk groups. We also used GSEA to investigate the signaling mechanisms of the risk model. Protein-protein interaction (PPI) network for DEGs was constructed with the Search Tool for the Retrieval of Interacting Genes (STRING) (https://string-db.org/) database. The quantity and quality of gene mutations were analyzed in two risk subgroups by the “Maftools” package of R.

### 2.8. Evaluation and Validation of the Prognostic Model

To evaluate the predictive value of the prognostic model, the time-dependent ROC curves were performed by the “survivalROC” R package. K-M analysis was also carried out to compare the OS among different risk groups. Besides, to ascertain whether the risk score could be considered as an independent factor to predict the prognosis of HCC patients, both risk score and clinicopathological information were incorporated in univariate and multivariate analyses. Based on the results of multivariate analyses, the nomogram prediction model was constructed to predict the survival probability of HCC patients at 1, 2, and 3 years. Calibration graphs were plotted to investigate whether the nomogram predicted survival rates were close to the actual survival rates. ROC curves were employed to assess the predictive effect of the nomogram model. To validate the accuracy of the prognostic model, the same analysis was also conducted in external validation cohorts (ICGC and GSE14520 cohorts).

### 2.9. Statistical Analysis

R software and SPSS were applied for all statistical analyses. The independent *t*-test was performed when continuous variables were normally distributed; otherwise, the Mann-Whitney *U* test was adopted. *χ*^2^ test or Fisher's exact test was performed to analyze categorical variables, as appropriate. A *p* value <0.05 was identified as statistically significant.

## 3. Results

### 3.1. Identification of Differentially Expressed Genes in TCGA

**Figure 1:**
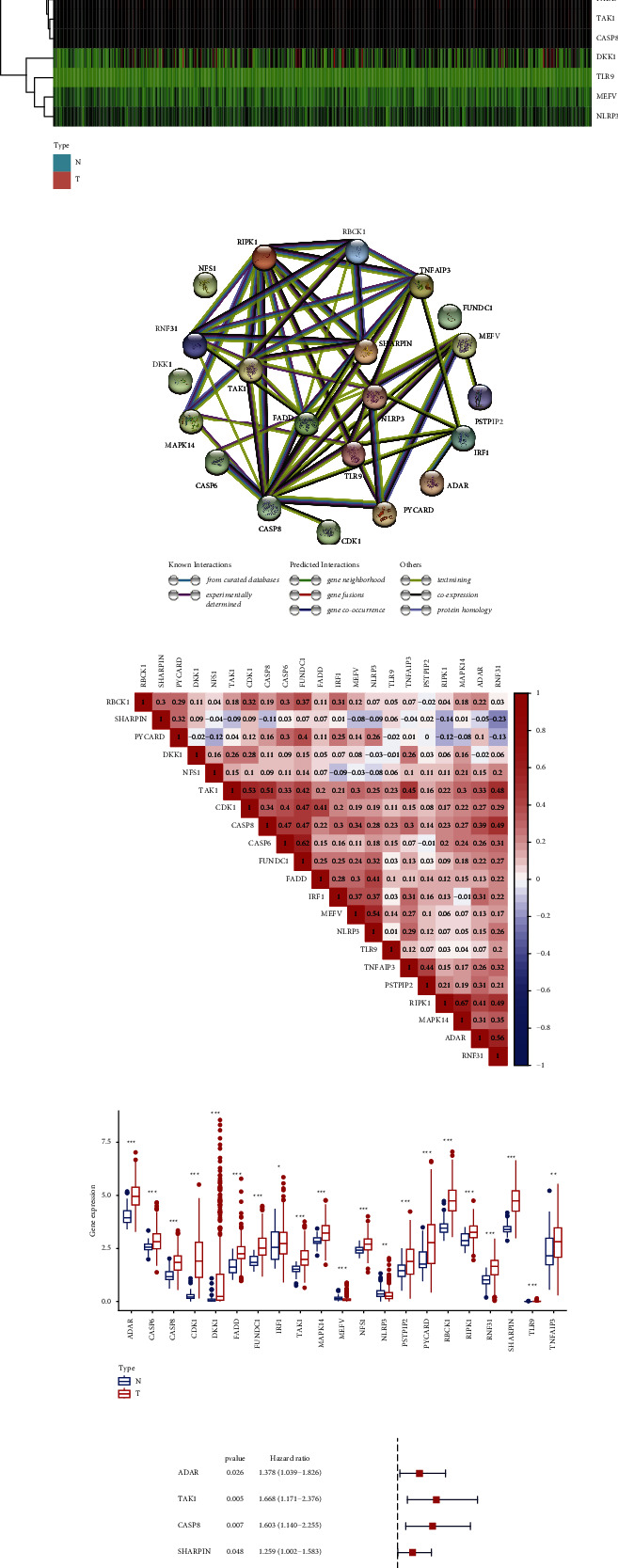
Identification of differentially expressed prognostic genes in TCGA. (a) Heatmap of 21 differentially expressed genes. (b) PPI network of 21 DEGs. (c) The correlation coefficient among the 21 DEGs. (d) Boxplot of the expression of 21 DEGs between HCC tissues and normal liver tissues. (e) Forest plots of 8 genes significantly correlated with the OS.

Based on the Wilcox test, 21 differentially expressed genes (DEGs) are picked out among 26 PANoptosis-related genes which are shown as heatmap (Figure 1(a)). The protein-protein interaction (PPI) network of the 21 DEGs was displayed in Figure 1(b), while their correlation coefficients were represented in Figure 1(c), which revealed the interactions among the DEGs. As shown in the boxplot, all DEGs except for NLRP3 were upregulated in HCC tissues (Figure 1(d)). Through univariate Cox regression, 8 DEGs were proved to be associated with overall survival (OS), which were risk factors for HCC patients (Figure 1(e)).

### 3.2. Building Clusters in HCC Based on PANoptosis-Related DEGs

**Figure 2:**
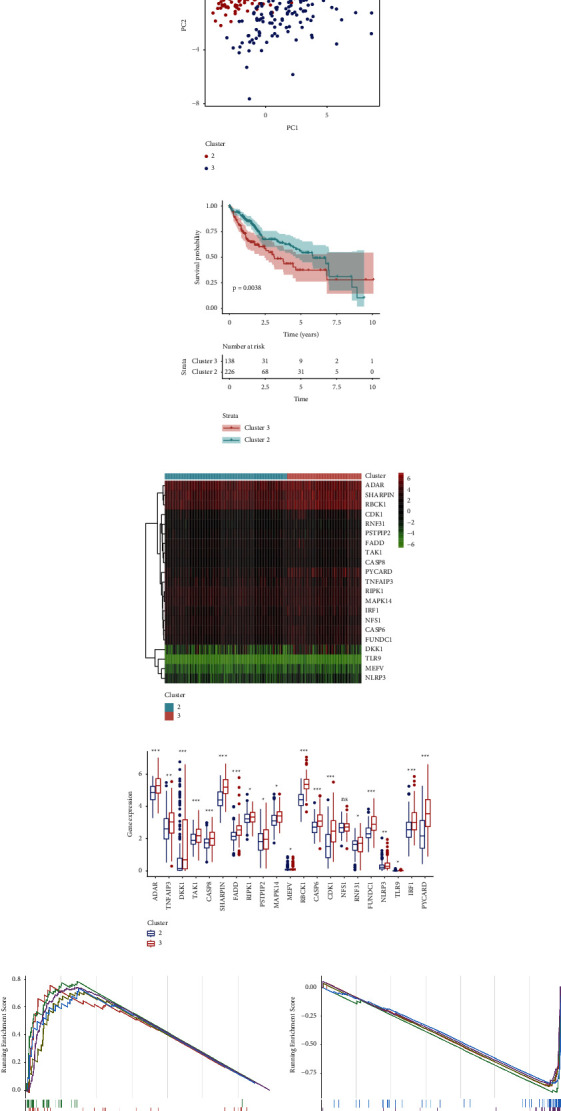
HCC clustering based on the differentially expressed PANoptosis-related genes. (a) Identification of three HCC clusters by consensus clustering analyses. (b) A good distinction between two clusters is shown in PCA. (c) KM analysis of the two clusters. (d) Heatmaps showed the relationship between the two clusters and DEGs' expression. (e) Boxplot of the expression of DEGs between the clusters. (f) GSEA for the KEGG pathways activated in clusters 3 and 2.

To explore the connection between DEGs expression and HCC subtypes, we performed consensus clustering analysis based on PANoptosis-related DEGs. It was found that when the clustering variable (*k*) was up to three, clustering analysis exhibited the optimal clustering stability, which was confirmed by the CDF curve and delta area (Figure 2(a) and Supplementary Figure S1(a)). Due to only 6 samples in cluster 1, it was hard to perform difference analysis between cluster 1 and other clusters, so clusters 2 and 3 were enrolled into the subsequent analysis. Besides, the same clustering also occurred in the validation cohorts (ICGC and GSE14520) (Supplementary Figures S1(b) and S1(e)), and the CDF curve confirmed the clustering (Supplementary Figures S1(c) and S1(f)), which suggested that the clustering of HCC cases based on PANoptosis-related DEGs was stable and reliable. As expected, there existed a satisfactory separation between clusters 2 and 3, as shown in PCA (Figure 2(b)). The baseline characteristics of the patients in the two clusters were described in Supplementary Table S2, which showed that patients in cluster 3 had higher mortality and less survival time (*p* < 0.05). KM analysis also revealed that HCC patients in cluster 3 had a significantly lower survival time than those in cluster 2 (*p*=0.0038) (Figure 2(c)). Similarly, it was also confirmed in validation cohorts that HCC subtypes clustered by DEGs were closely related to OS (Supplementary Figures S1(d) and S1(g)). In Figures 2(d) and 2(e), we found that cluster 3 had higher expression levels of the 20 DEGs that expected NFS1. To investigate the pathways activated in clusters 2 and 3, GSEA was also employed which unfolded that asthma, base excision repair, and mismatch repair displayed obvious activation in cluster 3, while arginine biosynthesis, fatty acid degradation, and retinol metabolism were remarkably activated in cluster 2 (Figure 2(f)). Moreover, cluster 3 had a higher level of inhibitory immune cells including Tregs and M0 macrophages with a low level of M2 macrophages and naive B-cells, indicating that the two clusters had different immune infiltration (Supplementary Figure S1(h)).

### 3.3. Construction and Validation of the PANoptosis-Related Prognostic Model

**Figure 3:**
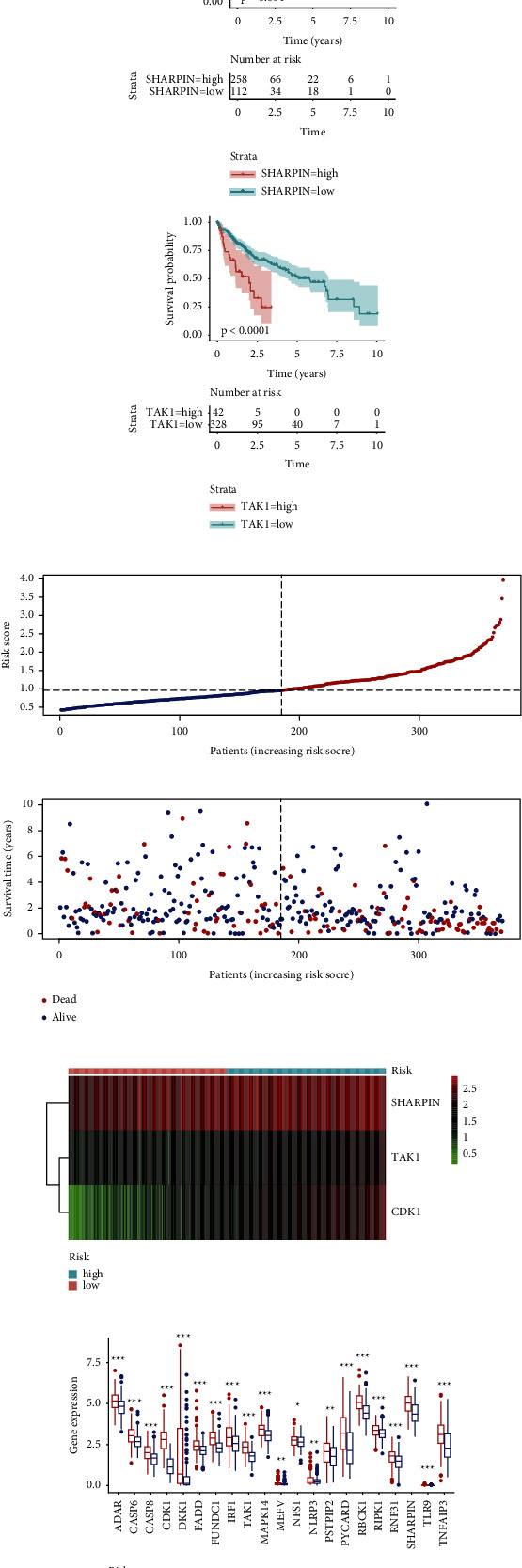
Construction of the prognostic model. (a) Coefficient profiles in the LASSO Cox regression model. (b) Validation for turning parameter selection in the LASSO Cox regression model. (c) KM curve of the three genes composed of the signature. (d) The distribution and median value of the risk scores in TCGA. (e) The distributions of OS status of HCC patients in TCGA. (f) Heatmap of the 3 genes among different risk groups. (g) Expression of DEGs among different risk groups. (h) PCA showed a good distinction among risk groups. (i) Comparison of risk score between the two clusters. (j) Sankey plot showed the correlation among clusters, risk groups, and survival status.

To improve the accuracy and reduce the complexity of the predictive signature, we used LASSO and Cox stepwise regression analyses to build a prognostic model (Figures 3(a) and 3(b)). After selection, 3 genes which had a negative correlation with a survival rate for HCC cases were enrolled into the formula of risk value calculation: risk score = (TAK1 × 0.3156) + (SHARPIN × 0.2255) + (CDK1 × 0.2624) (Figure 3(c)). Based on the median risk score, HCC cases were classified into high- and low-risk groups. The distributions of patients in the two groups were shown in Figures 3(d) and 3(e). Compared with the low-risk group, high-risk patients had a higher expression level of PANoptosis-related DEGs (Figures 3(f) and 3(g)).  Figure 3(h) showed that there existed an evident distinction between low- and high-risk groups. It was also found that cluster 3 had higher risk scores than cluster 2 (Figure 3(i)). The associations among cluster, risk groups, and survival status were shown in the Sankey diagram (Figure 3(j)).

### 3.4. Evaluation of the Tumor Microenvironment

**Figure 4:**
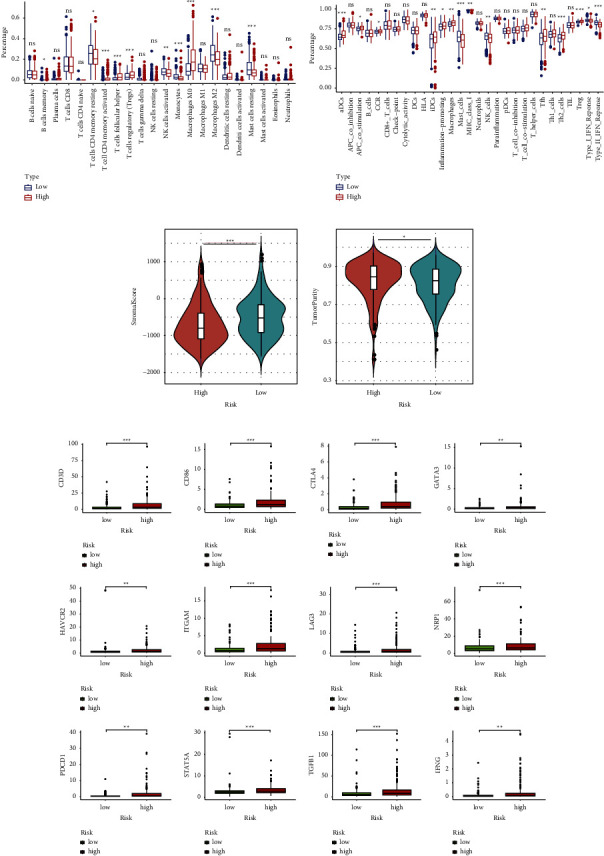
The characteristics of immune infiltration in different risk groups. (a) Comparison of immune cell abundance in different risk groups according to CIBERSORT analysis. (b) Relationship between the risk score and the 29 immune signatures according to ssGSEA analysis. (c-d) Correlation between the risk score and immune-related scores. (e) The relationship between immune cell markers and the risk score.

To investigate the correlation between the genes composed of the prognostic signature and immune cell infiltration, the TIMER2 online tool was utilized. CDK1 and TAK1 were positively correlated with the extent of B-cells, CD8^+^ T-cells, CD4^+^ T-cells, macrophage, neutrophil, and dendritic cells (*p* < 0.05) (Supplementary Figure S2(a)). The Wilcoxon test was employed to compare the distribution of 22 types of immune cells among risk subgroups. The boxplot revealed that memory B-cells, CD4 memory resting T-cells, CD4 memory activated T-cells, follicular helper T-cells, regulatory T-cells (Tregs), and M0 macrophages were upregulated in the high-risk group of TCGA cohort, while activated natural killer (NK) cells, monocytes, M2 macrophages, and resting mast cells were significantly downregulated (*p* < 0.05) (Figure 4(a)). The validation cohort presented similar results in which high-risk samples had a higher level of regulatory T-cells (Tregs) and M0 macrophages with lower extent of M2 macrophages (Supplementary Figures S2(b) and S2(c)).

Based on ssGSEA analysis, it was observed that 14 immune signatures displayed significant differences among risk groups (Figure 4(b)). As a result, high-risk patients had more Tregs with lower NK cells and type I IFN response, suggesting there were more immune-suppressive tumor microenvironments in high-risk population. Lower stromal scores and higher tumor purity scores were observed in high-risk population (Figures 4(c) and 4(d)). Similar to TCGA, the same results of stromal and tumor purity scores in ICGC and GSE14520 cohort are displayed in Supplementary Figures S2(d) and S2(e).

Correlation analysis was carried out to investigate the relationship between immune cell markers and the risk score. The expression of CD3D, CD86, CTLA4, GATA3, HAVCR2, IFNG, ITGAM, LAG3, NRP1, PDCD1, STAT5A, and TGFB1 was significantly higher in high-risk population, suggesting that the risk model based on PANoptosis-related genes was associated with HCC immune cell infiltration (Figure 4(e)).

### 3.5. The Efficacy of ICI Therapy in Different Risk Subgroups

**Figure 5:**
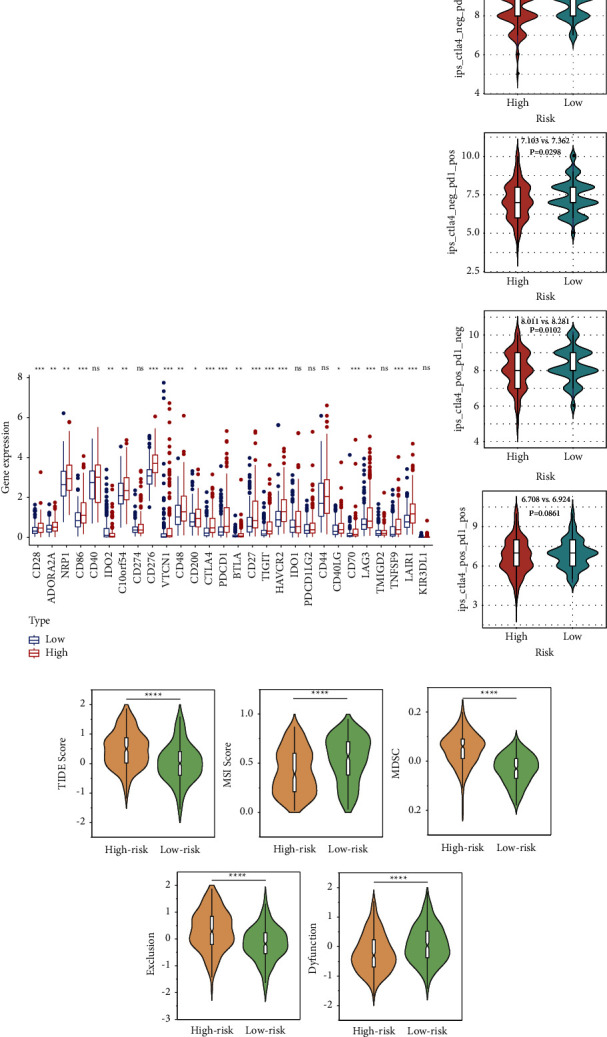
Immune checkpoints and the immunotherapeutic response indicator in different risk groups. (a) Expression of immune checkpoints among different risk groups. (b) IPS score between two groups. (c) The score of TIDE, MSI, MDSC, T-cell exclusion, and T-cell dysfunction between two groups.

To further compare the immune efficacy among different risk subgroups, the expression of immune checkpoint genes was enrolled into difference analysis. As a result, most immune checkpoints were upregulated in the high-risk subgroup, such as CD276, CTLA4, PDCD1, HAVCR2 (TIM3), LAG3, and TIGIT (Figure 5(a)). What is more, the expression of inhibitory immune checkpoint markers such as CTLA4, PDCD1, CD276, LAIR1, and HAVCR2 was also higher in high-risk population in the ICGC cohort (Supplementary Figure S3(a)). The IPS was an essential immune response indicator to predict the response to CTLA-4 and PD-1 inhibitors. Patients with a higher IPS value had a better response to PD-1 and CTLA-4 inhibitors, as the violin diagram showed that low-risk samples had higher IPS which suggested that HCC patients in the low-risk cohort could receive better immunotherapy efficacy (Figure 5(b)). Moreover, another immune response indicator TIDE was also compared among risk groups. It was found that patients in high-risk population had a higher score of TIDE, revealing that high-risk patients had more potential for immune escape and patients with a low risk were more suitable for immune checkpoint blockade, which was consistent with the aforementioned discover based on IPS (Figure 5(c)). A higher MSI score and a lower level of MDSC were observed in low-risk population, which also implied better response to immunotherapy. Additionally, we found that the high-risk group had a higher level of T-cell exclusion with a lower T-cell dysfunction score (Figure 5(c)). Similarly, the scores of TIDE, MSI, MDSC, T-cell exclusion, and T-cell dysfunction were significantly different in different risk groups in ICGC and GSE14520 cohort, which confirmed that high-risk patients had worse efficacy in receiving immunotherapy (Supplementary Figures S3(b) and S3(c)).

**Figure 6:**
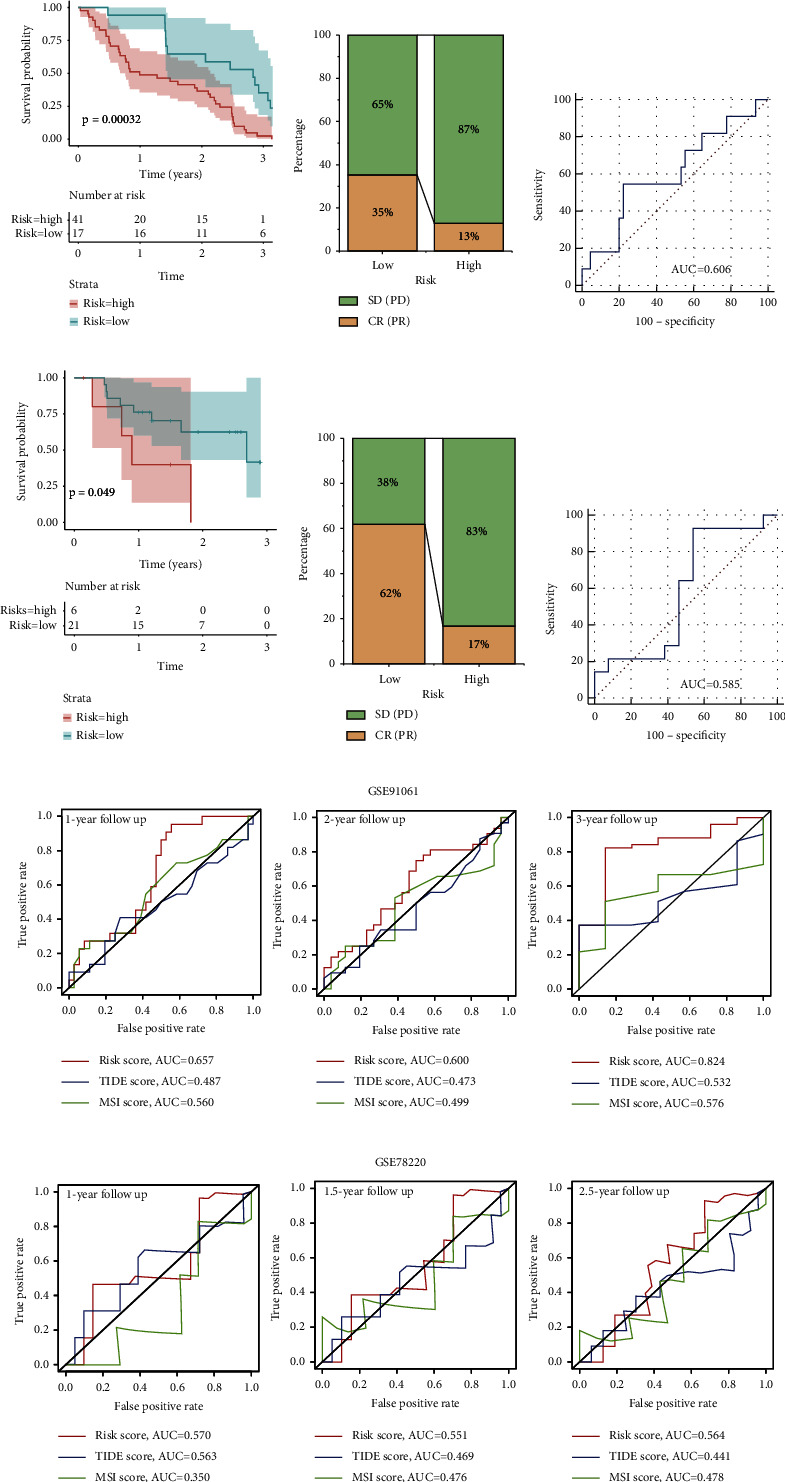
The prediction efficacy of immunotherapy based on the risk score. (a) KM curve for high- and low-risk subgroups in the anti-PD-L1 and anti-CTLA4 cohort (GSE91061). (b) The proportion of immune response (CR: complete response; PR: partial response; SD: stable disease; and PD: progressive disease) between two groups in GSE91061. (c) ROC analysis of the risk score on the response rate in GSE91061. (d) KM curve for high- and low-risk subgroups in the anti-PD-L1 (GSE78220). (e) The proportion of immune response (CR, PR, SD, and PD) between two groups in GSE78220. (f) ROC analysis of the risk score on the response rate in GSE78220. (g) ROC analysis of the risk score, MSI, and TIDE on OS at 1-, 2-, and 3-year follow-up in GSE91061. (h) ROC analysis of the risk score, MSI, and TIDE on OS at 1-, 1.5-, and 2.5-year follow-up in GSE78220.

To evaluate the potential clinical efficacy of immunotherapy in different risk groups, the prognostic signature was also employed in the anti-PD-L1 cohort (GSE78220) and anti-PD-L1 and CTLA4 cohort (GSE91061). A longer OS and better therapeutic response were observed in the low-risk group, indicating that low-risk population could benefit more from immune checkpoint inhibitor (ICI) therapy (Figures 6(a), 6(b), 6(d) and 6(e)). As the ROC curve showed, the risk score had a certain degree of predictive value for the immune response rate (Figure 6(c) and 6(f)). Moreover, ROC analysis of the risk score, TIDE, and MSI was implemented simultaneously to compare their predictive value for OS. The results showed that the AUCs for the risk score were greater than those for TIDE and MSI, suggesting that the risk score based on the PANoptosis-related prognostic model may be more suitable for prediction of prognosis of HCC patients under ICI therapy (Figure 6(g) and 6(h)).

### 3.6. Prediction and Validation of Chemotherapeutic Agents

**Figure 7:**
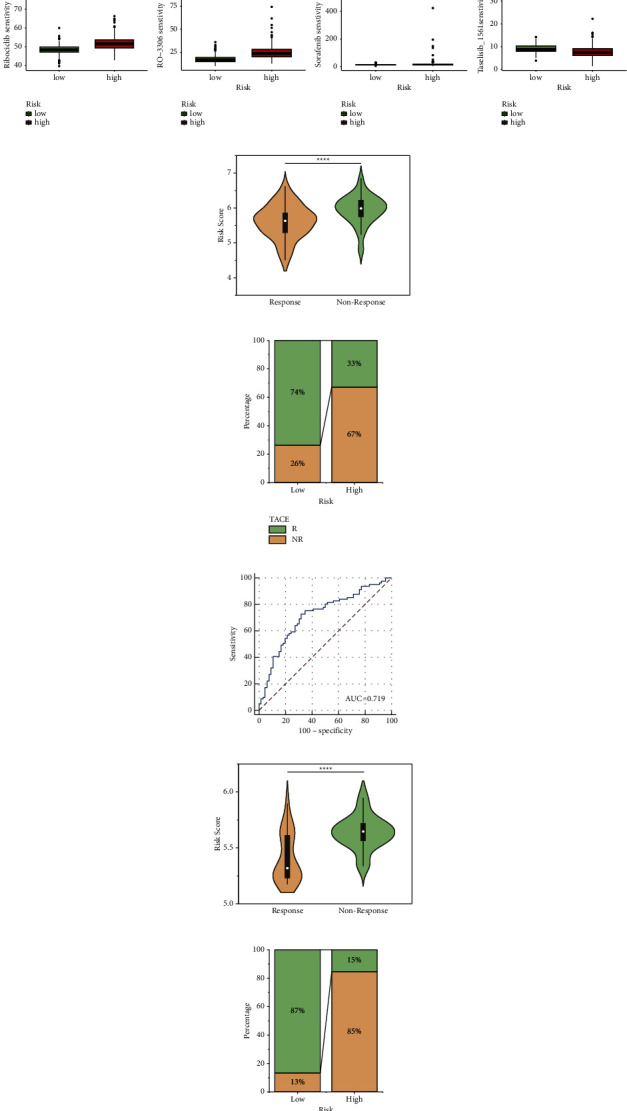
Efficacy prediction of chemotherapy drugs. (a) IC_50_ of 12 chemotherapy agents in different risk groups. (b) Comparison of the risk score between response and nonresponse subgroups in TACE cohort (GSE104580) (c) The proportion of chemotherapy response (response and nonresponse) in TACE cohort. (d) ROC analysis of the risk score on the response rate in TACE cohort. (e) Comparison of the risk score between response and nonresponse subgroups in the sorafenib cohort (GSE109211). (f) The proportion of chemotherapy response (response and nonresponse) in the sorafenib cohort. (g) ROC analysis of the risk score on the response rate in the sorafenib cohort.

In order to predict suitable drugs for HCC patients, the IC50 of 198 chemotherapeutic drugs was calculated and compared among risk subgroups based on PANoptosis-related prognostic signature. We found that the IC50 values of axitinib, AZD6482, AZD8055, BI-2536, JQ1, NU7441, linsitinb, PD0325901, ribociclib, RO-3306, and sorafenib were significantly lower in low-risk population, indicating that patients with a low risk were more sensitive to the 11 chemotherapeutic drugs (Figure 7(a)). By contrary, the PIK3CA inhibitor, taselisib, had a lower IC50 value in the high-risk group, indicating that high-risk patients had better drug sensitivity to taselisib (Figure 7(a)). Moreover, the expression of PIK3CA and its potential downstream molecule, AKT, were all found to be upregulated in the high-risk group (Supplementary Figure S4(a)), suggesting that there might be a close correspondence between the elevated sensitivity to taselisib and the high expression level of the target genes in the high-risk population.

TACE cohort (GSE104580) and sorafenib cohort (GSE109211) were utilized to verify the relationship between the risk score and sensitivity of drugs. A lower risk score was observed in TACE- and sorafenib-response groups, while low-risk population had a higher proportion of response to chemotherapeutic drugs (Figures 7(b), 7(c), 7(e) and 7(f)), revealing that low-risk HCC patients could obtain better efficacy from TACE and sorafenib therapy. The AUCs for response to TACE and sorafenib were 0.719 and 0.795 which indicated that the risk score had a good predictive value for predicting the response to TACE and sorafenib (Figures 7(d) and 7(g)). These results also confirmed the reliability of the prediction of chemotherapeutic responses in different risk groups, implying that low-risk HCC patients may benefit more from chemotherapy, while high-risk cases had a certain degree of chemotherapy resistance.

### 3.7. Molecular Characteristics of Different Risk Subgroups

**Figure 8:**
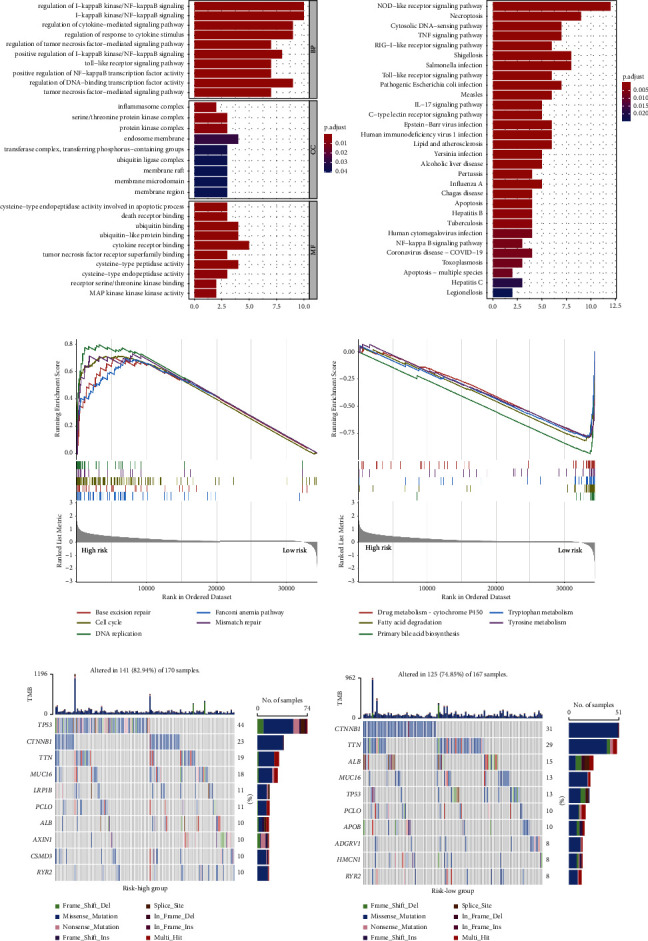
Molecular characteristics of different risk groups. (a) GO enrichment in TCGA database. (b) KEGG pathways in TCGA database. (c) GSEA in the high-risk group. (d) GSEA in the low-risk group. (e) The mutated genes (top 10) in different risk groups.

To elucidate the function differences among risk groups, we performed GO and KEGG enrichment analysis. According to the GO enrichment analysis, 21 DEGs upregulated in the high-risk group involved in the biological process of I-kappaB/NF-kappaB signaling, the cellular component of inflammasome complex, and the molecular function of cysteine-type endopeptidase/peptidase activity (Figure 8(a)). The KEGG enrichment analyses showed that these DEGs participated in the NOD-like receptor signaling pathway, TNF signaling pathway, IL-17 signaling pathway, and Toll-like receptor signaling pathway (Figure 8(b)). These pathways were closely related to tumor development and metastasis [13, 14], implying that the poor outcome of high-risk patients may result from aggressive cancer growth. To explore the signaling mechanisms of risk signature, GSEA was employed which was annotated by KEGG databases and included 48773 DEGs which were selected between high- and low-risk subgroups. It was found that high-risk population was mainly enriched in base excision repair (BER), cell cycle, mismatch repair, and other pathways (Figure 8(c)), while the low-risk group was mainly enriched in drug metabolism-cytochrome P450, fatty acid degradation, primary bile acid biosynthesis, tryptophan and tyrosine metabolism, and other pathways (Figure 8(d)). Three representative genes (NEIL2, OGG1, and APEX1) of BER were upregulated in high-risk population [15], confirming that patients with a high risk had more enriched BER activity (Supplementary Figure S4(b)).

As the gene mutation analysis showed, the mutation of the TP53 gene was more common in the high-risk subgroup, while low-risk population had higher proportion of the mutation of CTNNB and TTN genes (Figure 8(e)). The relationship between the risk score and TMB was investigated which showed that the risk score was not significantly correlated with TMB (Supplementary Figure S4(c)).

### 3.8. Estimation and Validation of the Prognostic Model

**Figure 9:**
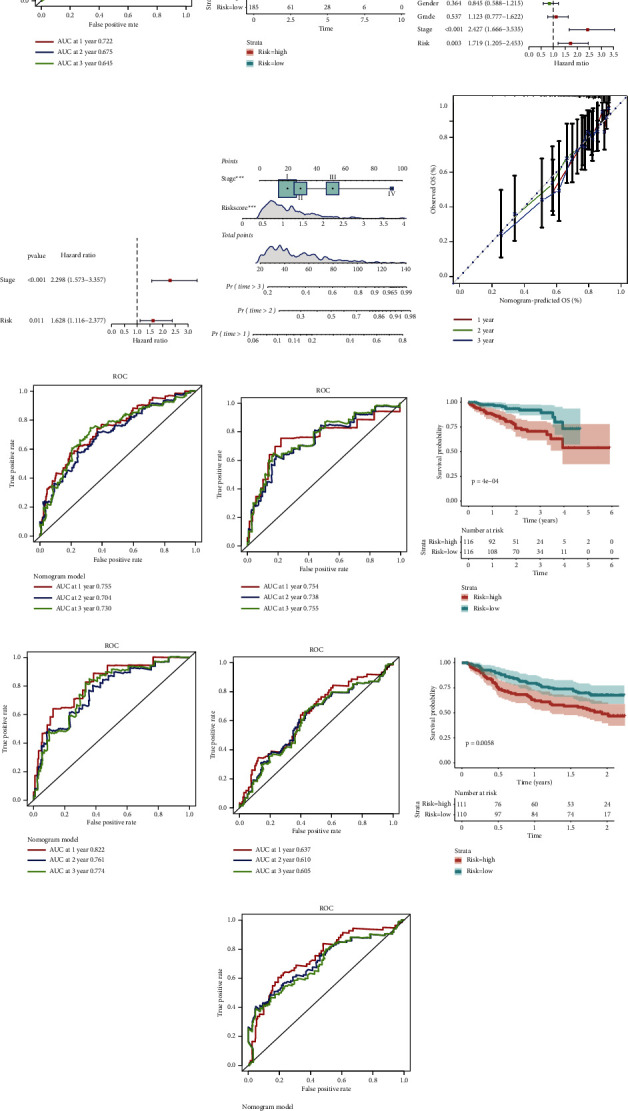
Evaluation and validation of the prognostic signature. (a) AUC of time-dependent ROC curves in TCGA at 1 year, 2 years, and 3 years. (b) K-M analysis of the risk score in TCGA. (c, d) Forest plot of the univariate and multivariate regression analysis in TCGA cohort. (e) Nomogram based on the risk score and other clinical features for predicting 1- to 3-year OS in TCGA. (f) Calibration graphs investigated that whether the nomogram predicted survival rates were close to the actual survival rates. (g) ROC analysis of the nomogram model in TCGA (h) AUC of time-dependent ROC curves in ICGC at 1 year, 2 years, and 3 years. (i) K-M analysis of the risk score in the ICGC cohort. (j) ROC analysis of the nomogram model in ICGC. (k) AUC of time-dependent ROC curves in GSE14520 at 1 year, 2 years, and 3 years. (l) K-M analysis of the risk score in the GSE14520 cohort. (m) ROC analysis of the nomogram model in GSE14520.

As shown in the ROC curves, the areas under the curves (AUCs) for one-, two-, and three-year survival were 0.722, 0.675, and 0.645, respectively (Figure 9(a)). The KM curve was drawn to reveal that a lower survival probability occurred in high-risk population (Figure 9(b)). To test the capacity of the signature as an independent prognostic indicator which was different from traditional clinical elements, the variables of the risk score, age, AFP, gender, grade, and stage were enrolled into Cox regression analysis. The results uncovered that the risk score and TNM stage were independent risk factors for OS in TCGA (Figure 9(c) and 9(d)). Thus, we used the risk score and stage to develop a nomogram model (Figure 9(e)). To assess the predictive accuracy of the nomogram, calibration plots were drawn. As exhibited in Figure 9(f), the nomogram-predicted survival probabilities were remarkably close to the actual survival outcomes. To evaluate the predicted value of the nomogram model, the ROC curve was plotted which showed that the nomogram model had a larger AUC value at 1 year, 2 year, and 3 years than the prognostic model based on the risk score (Figure 9(g)), indicating that uniting the risk score and TNM stage to build the nomogram model to predict OS was more accurate and had a greater predictive value.

To verify these results, the ROC curve, KM analysis, and nomogram were also performed in the two independent validation cohorts. In ICGC, AUCs were 0.754, 0.738, and 0.755 at 1 year, 2 years, and 3 years, respectively, while the AUCs were 0.637, 0.610, and 0.705 at 1 year, 2 years, and 3 years in GSE14520 (Figures 9(h) and 9(k)), suggesting that the risk score was a reliable prognostic indicator. KM analysis uncovered that high-risk patients had higher mortality in both ICGC and GSE14520 cohorts (Figures 9(i) and 9(l)). Cox regression analysis and nomogram in validation cohorts confirmed that the TNM stage and the risk score were valuable independent prognostic factors for OS (Supplementary Figures S5(a), S5(b), S5(d), and S5(e)). The calibration plots implied the high accuracy of this nomogram model (Supplementary Figures S5(c) and S5(f)). Figures 9(j) and 9(m) also suggested that the nomogram model based on the TNM stage and risk score had a better predictive effect with a greater AUC value in validation cohorts, which was in accordance with the results of TCGA.

## 4. Discussion

PANoptosis, a newly recognized pathway of programmed inflammatory cell death driven by PANoptosome, combined the main features of pyroptosis, apoptosis, and necroptosis [16]. In recent years, PANoptosis had become a research hotspot in malignant tumors. ADAR1 facilitated the proliferation of tumor cells by inhibiting PANoptosis [17]. NFS1 deficiency could trigger PANoptosis to increase the sensitivity of colorectal cancer cells to oxaliplatin [18]. Karki et al. also found that IRF1 regulated PANoptosis to suppress the growth of colorectal cancer [19]. However, the role of PANoptosis in HCC still remained unclear. Previous studies reported there existed a close connection between PANoptosis and innate immune, especially inflammatory immune response [20]. It was found that ZBP1, the component of PANoptosome, was a critical innate immune sensor, while the regulation of PANoptosis by RIPK1 was essential for inflammatory immune responses. In addition, it was also reported that sorafenib resistance was closely related to the inhibition of pyroptosis, apoptosis, and necroptosis [10, 11, 21]. Thus, using PANoptosis-related genes to predict the efficacy of immunotherapy and sorafenib treatment might be a good option.

Due to the substantial immunosuppressive activity in HCC, the immune-based treatments for this difficult-to-treat cancer were promising [22]. Currently, immunotherapy has become a non-negligible treatment option for HCC patients, but the effect of immunotherapy is closely related to the tumor microenvironment (TME) [23, 24]. In this study, patients with a high risk had a higher level of immunosuppressive cells such as MDSCs, M0 macrophages, and Tregs, with lower infiltration of cytotoxic immune cells, such as NK cells. Treg cells suppress antitumor immunity through suppressing antigen presenting cells (APCs) to further prevent activation of T-cells, which facilitate immune evasion [25]. Previous studies reported that Tregs predicted poor survival in HCC patients [26]. Similarly, MDSCs induced immune escape through expressing immunosuppressive factors [27]. A high level of MDSCs was also closely related to poor prognosis in HCC [28]. Thus, it was no surprise that the TIDE score was higher in high-risk population, suggesting that high-risk patients had greater immune escape potentiality and worse response to immunotherapy. The worse prognosis of high-risk patients might result from the immunosuppressive TME.

Inhibitory immune checkpoints (ICPs) could reduce the immunogenicity of tumor cells, thereby avoiding immune surveillance [28]. What is more, inhibitory immune checkpoints could stimulate Tregs differentiation, while Tregs could also express inhibitory ICPs especially CTLA4. The close cooperation between Tregs and inhibitory ICPs confered a strong immunosuppressive activity and caused a worse effect for immunotherapy [29]. Here, we found that high-risk patients had a higher level of inhibitory immune checkpoints including CTLA4, PDCD1, TIGHT, LAG3, and TIM3, which represented greater immune evasion and worse immunotherapeutic effects in high-risk population. Lower response to immunotherapy in high-risk population might be due to immunosuppression and immune evasion.

In our study, the higher level of IPS and MSI and the lower TIDE score in the low-risk subgroup revealed that patients with a low risk had better response to PD-1 and CTLA-4 inhibitor therapy. In the cohorts of anti-PD-1 and anti-CTLA-4, low-risk patients had a lower mortality rate and better therapeutic response, suggesting that patients with a low risk could acquire a better curative effect of anti-PD-1 and anti-CTLA-4 treatment. Compared with TIDE and MSI, the risk score had larger AUCs, revealing that the predictive value of the risk score for ICI therapy was greater than TIDE and MSI, and the risk score based on PANoptosis-related prognostic signature might be a better predictor of OS for HCC patients under ICI therapy.

The lower IC50 values denoted the greater sensibility to chemotherapeutic drugs. Here, we identified 12 significant chemotherapeutic drugs for HCC treatment based on IC50 and found that almost all of them had a lower IC50 value in low-risk population expect for taselisib. The results indicated that chemotherapeutic agents including axitinib, AZD6482, AZD8055, BI-2536, JQ1, NU7441, linsitinb, PD0325901, ribociclib, RO-3306, and sorafenib could provide good efficacy for low-risk patients, suggesting that low-risk patients might have a better response to chemotherapy. As for patients in the high-risk subgroup, the PIK3CA inhibitor, taselisib, had better efficacy and provided more benefit. Besides, we found that PIK3CA and its downstream molecule, AKT, were upregulated in the high-risk group, revealing that high-risk cases had elevated activity of the PI3K/AKT pathway. Kaklamani et al. reported that individuals with high activity of the PIK3CA pathway were more sensitive to taselisib [30]; therefore, taselisib may be more suitable for patients in high-risk population. As for intermediate-stage HCC patients, TACE was the standard therapy which achieved a good effect [31]. Sorafenib was the effective first-line therapy for advanced HCC cases, but the drug resistance to sorafenib was becoming more common [32]. Here, we utilized cohorts of TACE treatment and sorafenib therapy for HCC patients to explore the capability of the risk score to predict the efficacy of TACE and sorafenib therapy. The results indicated that HCC patients with a low risk had a better response to TACE and sorafenib. Moreover, the AUCs of the risk score had a great value, which implied that the risk score had a satisfactory predictive value for predicting the response to TACE and sorafenib. Therefore, HCC patients with a low risk should receive TACE and sorafenib therapy, and the risk score based on the PANoptosis-related prognostic model could be a predictive marker for TACE treatment and sorafenib therapy to guide clinical practice.

In addition, GSEA analysis revealed that high-risk cases had increased base excision repair (BER) activity. BER could preserve the integrity of DNA caused by cellular oxidative stress and exogenous insults [33]. However, tumor cells generally utilized BER to repair DNA damage and reduced the efficacy of radiotherapy and chemotherapy. High activity of BER was one of the main reasons for chemoresistance [34]. Recently, targeting BER enzymes had achieved a good effect in clinical trials [35, 36]. Thus, chemotherapy in combination with BER enzyme inhibitors might be a better option for cancer with strong chemotherapy resistance. In our study, the low sensitivity to chemotherapeutic drugs in the high-risk group might be partly attributed to the elevated activity of BER. Combination chemotherapy with targeting BER may be a good choice for the treatment of HCC patients with a high risk, which needs further research.

This signature was made up of three genes, TAK1, CDK1, and SHARPIN. Transforming growth factor beta-activated kinase 1 (TAK1), a fundamental component of innate and adaptive immune signaling, acted as a master switch for PANoptosis quiescence [16]. Inhibition of TAK1 generally caused the activation of PANoptosis and facilitated inflammatory immune responses [37]. It was reported that TAK1 was essential for survival and maintenance of peripheral T-cells, and TAK1 could regulate NK cell-mediated cytotoxicity [38]. In HCC, TAK1 facilitated tumor metastasis and progression, indicating an unfavorable outcome [39, 40]. As for cyclin dependent kinase 1 (CDK1), playing a crucial role in the control of the cell cycle, could regulate PANoptosis negatively through the ZBP1-dependent way [41]. Previous studies revealed that CDK1 which could induce tumor proliferation was also positively correlated with CD4⁺ T-cells and CD8⁺ T-cells in HCC [42, 43]. Besides, PANoptosome activation was also inhibited by SHARPIN (SHANK-associated RH domain interactor) [44]. Tanaka et al. found that SHARPIN promoted HCC progression via the Wnt/*β*-catenin pathway [45]. Similarly, recent studies uncovered that SHARPIN could regulate the pathways of NF-*κ*B and interferon antiviral, which was regarded as a novel modulator of immune responses [46]. Thus, there was a close relationship between the tumor immune microenvironment and this prognostic signature based on TAK1, CDK1, and SHARPIN. Moreover, it was noteworthy that the three genes could enhance the sorafenib resistance and therefore reduced the efficacy of chemotherapy [47–49]. These findings were in accordance with the results of IC50, suggesting that high-risk patients might benefit less from sorafenib treatment. In summary, this prognostic signature was closely related to immune response and tumor progression.

The functional analysis revealed that there were more tumor metastasis-related pathways in the high-risk subgroup. TP53 mutation acted as drivers of tumor progression which were involved in the tumor metastasis-related signals [50]. A higher level of TP53 mutation and immunosuppressive cells were displayed in high-risk population. In this study, a shorter survival time was observed in the high-risk group, which might result from immunosuppressive TME and more metastasis-related pathways. To better apply the signature to the clinical practice, we evaluated its forecast effect. The great value of AUCs proved the signature had well-prediction efficiency for OS. The nomogram model based on the risk score displayed a better predictive effect, suggesting that combining the risk score and stage may predict prognosis with higher accuracy. The abovementioned prognostic analysis was also employed in validation cohorts which showed good results, indicating that the prognostic signature we developed had a well-predicted value with high reliability and accuracy.

## 5. Conclusions

Our study demonstrated that the prognostic signature based on PANoptosis-related genes had a well-predictive efficacy for OS in HCC. HCC patients in low-risk population could acquire more benefit from ICI, TACE, and sorafenib therapy. Thus, ICI, TACE, and sorafenib therapy were more suitable for low-risk patients, while taselisib or combination of BER inhibitors and chemotherapy might be a better option for patients with a high risk. These findings suggested that this signature could be used as a biomarker to predict the efficacy of ICI therapy and chemotherapy to aid clinical therapeutic decision-making, which needs further research.

## Data Availability

The data of TIDE, MSI, and MDSC were acquired online (http://tide.dfci.harvard.edu/). IPS was obtained from Cancer Immunome Atlas (TCIA, https://tcia.at/). The RNA-Seq data and corresponding clinical information of HCC specimens were downloaded from TCGA database (https://portal.gdc.cancer.gov/), ICGC database (https://dcc.icgc.org/), and GSE14520 database (https://ncbi.nlm.nih.gov/geo/query/acc.cgi?acc=GSE14520). The sorafenib cohort was downloaded from the GEO database (https://ncbi.nlm.nih.gov/geo/query/acc.cgi?acc=GSE109211), and TACE cohort was downloaded online (https://ncbi.nlm.nih.gov/geo/query/acc.cgi?acc=GSE104580).

## Supplementary Materials

Supplementary Materials Figure S1: Validation of the HCC cluster in validation cohorts. Figure S2: The characteristics of immune infiltration in different risk groups in validation cohorts. Figure S3: Immune checkpoints and an immunotherapeutic response indicator in validation cohorts. Figure S4: Some important indicators in different risk groups. Figure S5: Nomogram model in validation cohorts. Table S1: 26 PANoptosis-related genes; Table S2: The clinical characteristics of HCC patients in different groups.
