# Supplementary material for: Identification of PANoptosis-Based Prognostic Signature for Predicting Efficacy of Immunotherapy and Chemotherapy in Hepatocellular Carcinoma
Source: Genet Res (Camb). 2023 Jun 5;2023:6879022. doi: 10.1155/2023/6879022 (PMC10260314; doi:10.1155/2023/6879022)
Supplement: Supplementary Materials — Figure S1: Validation of the HCC cluster in validation cohorts. Figure S2: The characteristics of immune infiltration in different risk groups in validation cohorts. Figure S3: Immune checkpoints and an immunotherapeutic response indicator in validation cohorts. Figure S4: Some important indicators in different risk groups. Figure S5: Nomogram model in validation cohorts. Table S1: 26 PANoptosis-related genes; Table S2: The clinical characteristics of HCC patients in different groups. [file 6879022.f1.zip › Supplementary Figures.pdf]

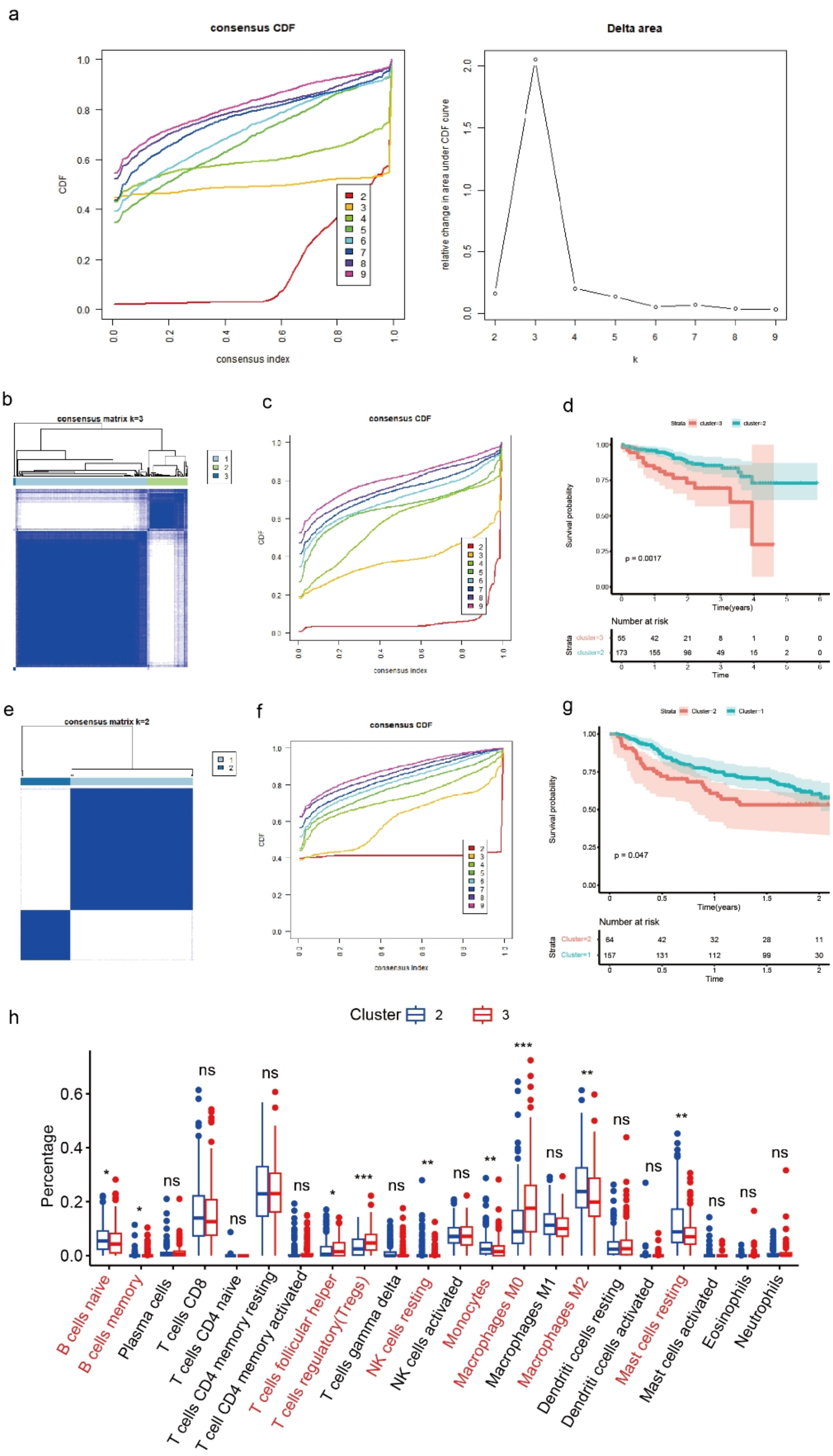

Supplementary Figure S1. Validation of HCC Cluster in validation cohorts. (a) Consensus CDF and delta area in TCGA to verify the HCC cluster in TCGA. (b) Identification of three HCC clusters in ICGC. (c) Consensus CDF in ICGC to verify the HCC cluster. (d) KM curve showed there exist different in OS between clusters in ICGC. (e) Identification of the HCC clusters in GSE14520. (f) Consensus CDF in GSE14520 to verify the HCC cluster. (g) KM curve showed there exist different

in OS between clusters in GSE14520. (h) Comparison of immune cell abundance in different clusters according to CIBERSORT analysis.

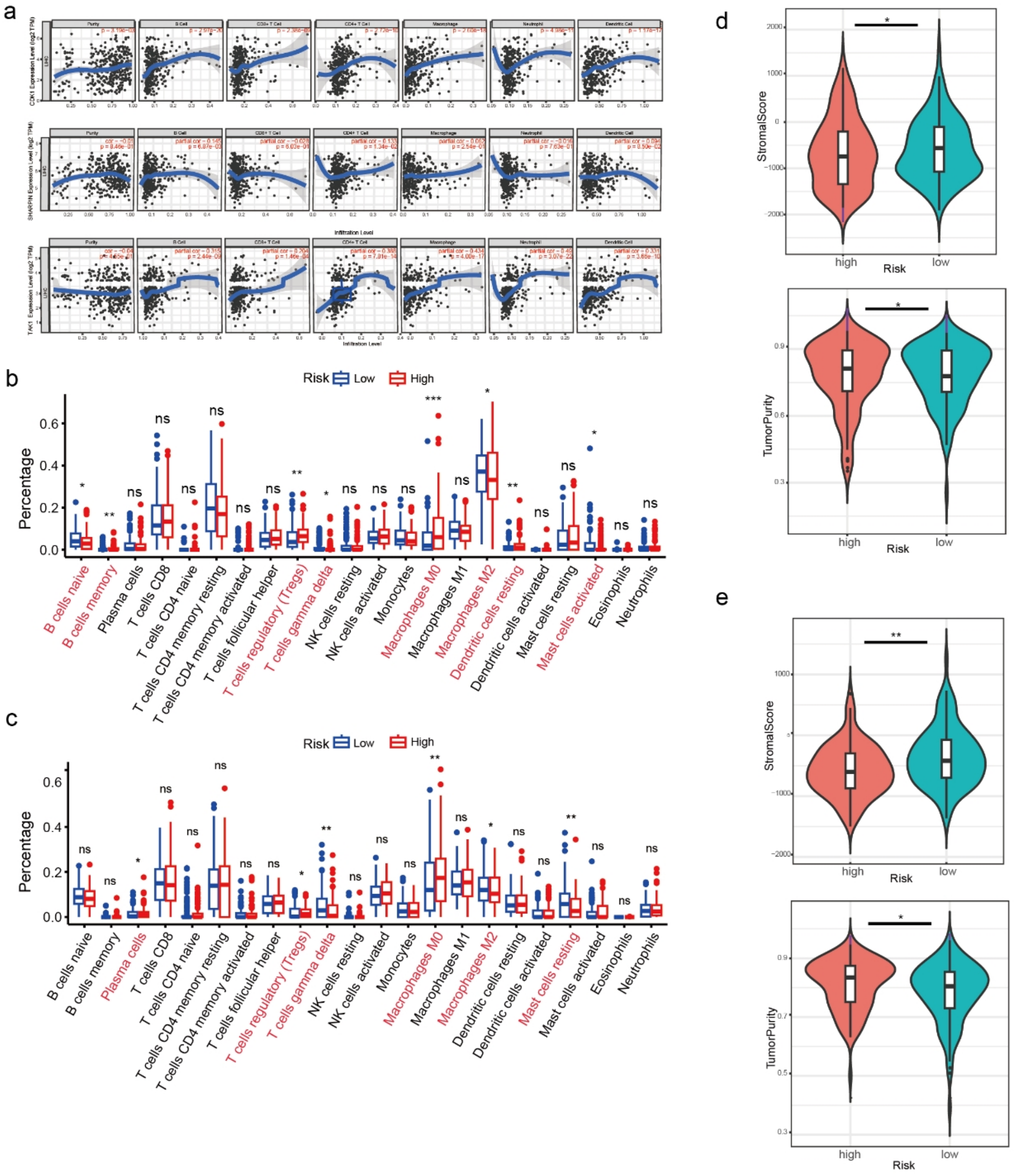

Supplementary Figure S2. The characteristics of immune infiltration in different risk groups in validation cohorts. (a) Relationship between the 3 genes (TAK1, SHARPIN, CDK1) and immune cells. (b) Comparison of immune cell abundance in different risk groups according to CIBERSORT analysis in ICGC. (c) Relationship between risk score and the 29 immune signatures according to ssGSEA analysis in ICGC. (d-e) Correlation between risk score and immune-related scores in ICGC and GSE14520 cohort.

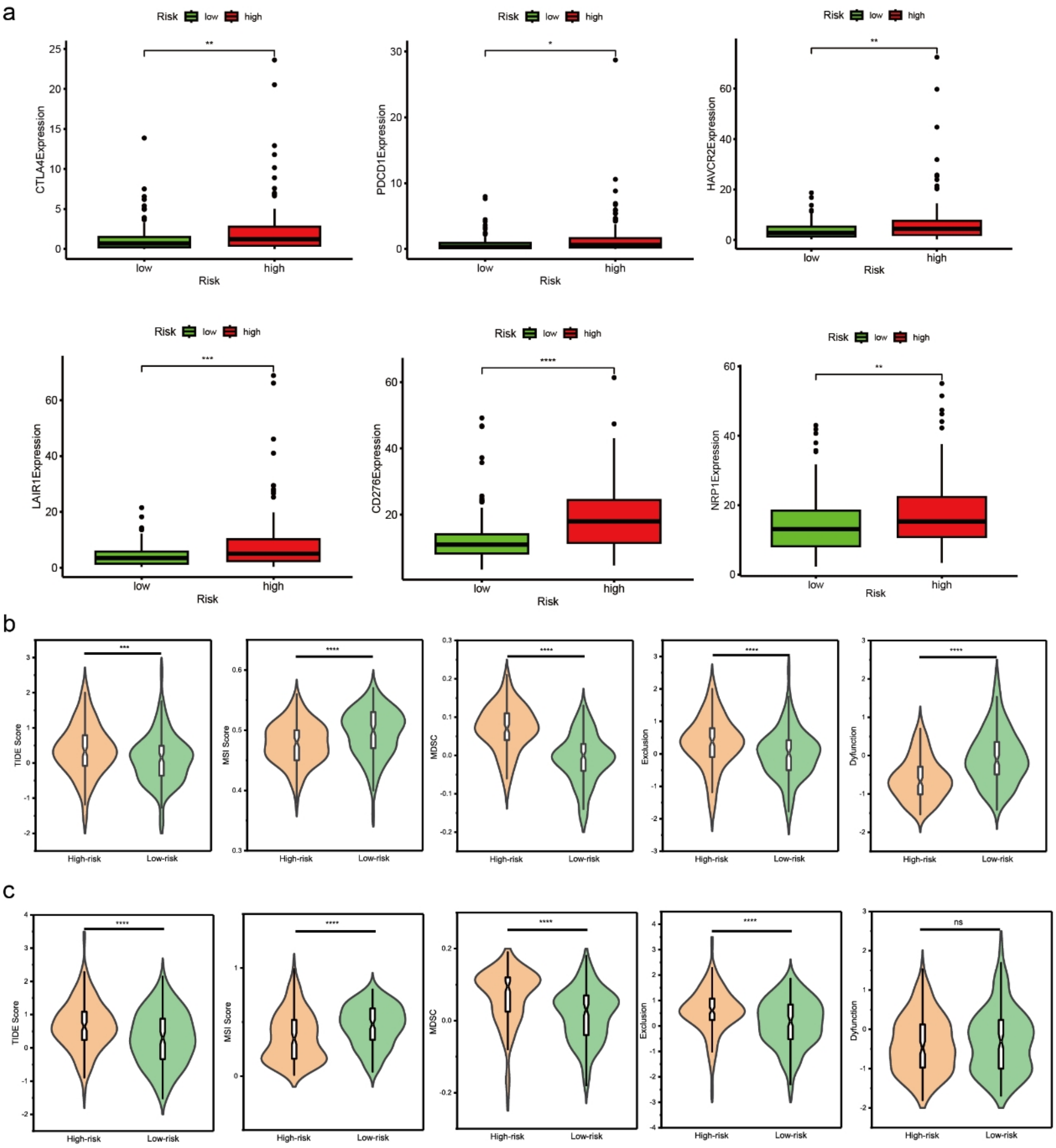

Supplementary Figure S3. Immune checkpoints and immunotherapeutic response indicator in validation cohorts. (a) Expression of inhibitory immune checkpoints in ICGC. (b-c) The immunotherapeutic response indicators in different risk groups in ICGC and GSE14520 cohort.

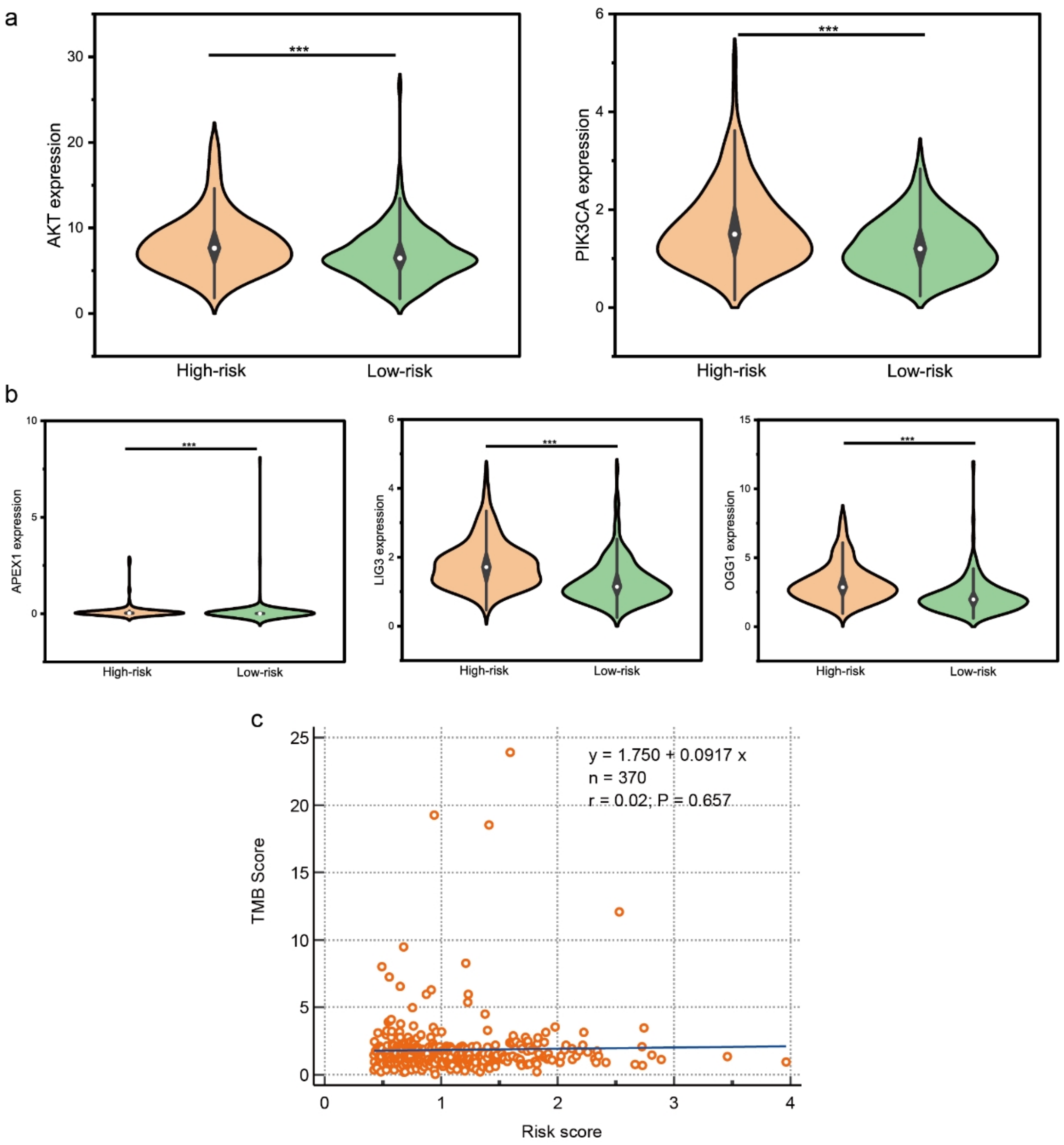

Supplementary Figure S4. Some important indicators in different risk groups. (a) The expression of AKT and PIK3CA. (b)The expression of key genes of BER (APEX1, LIG3 and OGG1). (c) The relationship between risk score and TMB.

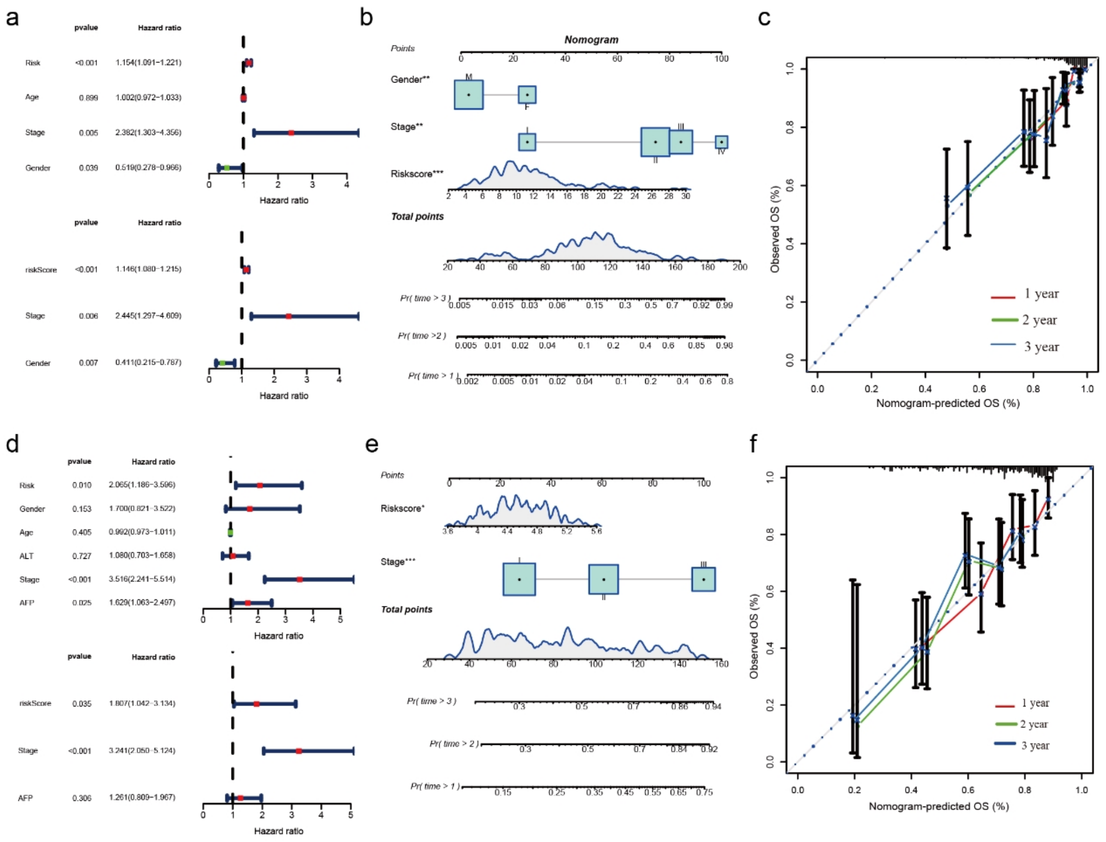

Supplementary Figure S5. Nomogram model in validation cohorts. (a) Forest plot of the univariate and multivariate regression analysis in ICGC. (b) Nomogram based on risk score and other clinical features in ICGC. (c) Calibration graphs investigated whether the nomogram predicted survival rates were close to the actual survival rates in ICGC. (d) Forest plot of the univariate and multivariate regression analysis in the GSE14520 cohort. (e) Nomogram based on risk score and other clinical features for predicting 1- to 3-year OS in the GSE14520 cohort. (f) Calibration graphs investigated whether the nomogram predicted survival rates were close to the actual survival rates in the GSE14520 cohort.
