# Supplementary material for: Identification of PANoptosis-Based Prognostic Signature for Predicting Efficacy of Immunotherapy and Chemotherapy in Hepatocellular Carcinoma
Source: Genet Res (Camb). 2023 Jun 5;2023:6879022. doi: 10.1155/2023/6879022 (PMC10260314; doi:10.1155/2023/6879022)
Supplement: Supplementary Materials — Figure S1: Validation of the HCC cluster in validation cohorts. Figure S2: The characteristics of immune infiltration in different risk groups in validation cohorts. Figure S3: Immune checkpoints and an immunotherapeutic response indicator in validation cohorts. Figure S4: Some important indicators in different risk groups. Figure S5: Nomogram model in validation cohorts. Table S1: 26 PANoptosis-related genes; Table S2: The clinical characteristics of HCC patients in different groups. [file 6879022.f1.zip › Supplementary Table S1.docx]

Supplementary Table S1: 26 PANoptosis-related genes

| **PANoptosis-related genes** | | | | |
| --- | --- | --- | --- | --- |
| ZBP1 | NLRP3 | CASP1 | ADAR | STING1 |
| RIPK1 | PYCARD | PSTPIP2 | AIM2 | RBCK1 |
| RIPK3 | FADD | RNF31 | NFS1 | TNFAIP3 |
| CASP8 | CASP6 | SHARPIN | IRF1 | TAK1 |
| FUNDC1 | DKK1 | CDK1 | MEFV | TLR9 |
| MAPK14 |  |  |  |  |
