# Supplementary material for: Identification of PANoptosis-Based Prognostic Signature for Predicting Efficacy of Immunotherapy and Chemotherapy in Hepatocellular Carcinoma
Source: Genet Res (Camb). 2023 Jun 5;2023:6879022. doi: 10.1155/2023/6879022 (PMC10260314; doi:10.1155/2023/6879022)
Supplement: Supplementary Materials — Figure S1: Validation of the HCC cluster in validation cohorts. Figure S2: The characteristics of immune infiltration in different risk groups in validation cohorts. Figure S3: Immune checkpoints and an immunotherapeutic response indicator in validation cohorts. Figure S4: Some important indicators in different risk groups. Figure S5: Nomogram model in validation cohorts. Table S1: 26 PANoptosis-related genes; Table S2: The clinical characteristics of HCC patients in different groups. [file 6879022.f1.zip › Supplementary Table S2.docx]

**Table S2.** The clinical characteristics of HCC patients in different groups.

| **Characteristics** | **Signature** | | **P** | **Cluster** | | **P** |
| --- | --- | --- | --- | --- | --- | --- |
|  | **High risk**  **(n=185)** | **Low risk**  **(n=185)** |  | **Cluster 2**  **(n=203)** | **Cluster 3**  **(n=146)** |  |
| **Age (>60 year)** | 85(45.9%) | 116(62.7%) | 0.001 | 110(54.2%) | 87(54.0%) | 0.977 |
| **Gender** | | | | | | |
| **Female** | 61(33.0%) | 60(32.4%) | 0.912 | 64(31.5%%) | 54(33.5%%) | 0.684 |
| **Male** | 124(67.0%) | 125(67.6%) |  | 139(68.5%) | 107 (66.5%) |  |
| **Grade** | | | | | | |
| **G1+G2** | 90(48.6%) | 142(76.8%) | <0.001 | 149(73.4%) | 81(50.3%) |  |
| **G3+G4** | 93(50.3%) | 40(21.6%) |  | 52(25.6%) | 77(47.8%) | <0.001 |
| **Unknow** | 2(1.1%) | 3(1.6%) |  | 2(1.0%) | 3(1.9%) |  |
| **T** | | | | | | |
| **T1+T2** | 131(70.8%) | 146(78.9%) | 0.072 | 157(77.3%) | 116(72.0%) | 0.247 |
| **T3+T4** | 54(29.2%) | 39(21.1%) |  | 46(22.7%) | 45(28.0%) |  |
| **M** | | | | | | |
| **M0** | 137(74.1%) | 129(69.7) |  | 145(71.4%) | 116(72.0%) |  |
| **MI** | 1(0.5%) | 3(1.6) | 0.449 | 1(0.5%) | 3(1.9%) | 0.434 |
| **Unknow** | 47(25.4%) | 53(28.6) |  | 57(28.1%) | 42(26.1%) |  |
| **N** | | | | | | |
| **N0** | 133(71.9%) | 120(64.9%) |  | 139(68.5%) | 110(68.3%) |  |
| **NI** | 3(1.6%) | 1(0.55) | 0.539 | 2(1.0%) | 2(1.2%) | 0.841 |
| **Unknow** | 49(26.5%) | 64(34.6%) |  | 62(30.5%) | 49(30.4%) |  |
| **Stage** | | | | | | |
| **I+II** | 121(65.4%) | 135(73.0%) | 0.151 | 147(72.4%) | 105(65.2%) | 0.336 |
| **III+IV** | 53(28.6%) | 37(20.0%) |  | 44(21.7%) | 44(27.3%) |  |
| **Unknown** | 11(5.9%) | 13(7.0%) |  | 12(5.9%) | 12(7.5%) |  |
| **AFP** | | | | | | |
| **>200ng/ml** | 57(30.8%) | 19(10.3%) | <0.001 | 21(10.3%) | 52(32.3%) |  |
| **≤200ng/ml** | 77(41.6%) | 124(67.0%) |  | 132(65.0%) | 69(42.9%) | <0.001 |
| **Unknow** | 51(27.6%) | 42(22.7%) |  | 50(24.6%) | 40(24.8%) |  |
| **Survival status** | | | | | | |
| **OS time (year)** | 1.170(0.732-2.097) | 1.748(1.011-3.673) | <0.001 | 1.729(1.079-3.38) | 1.137(0.599-2.366) | <0.001 |
| **Deceased** | 70(37.8%) | 56(30.3%) | 0.008 | 58(28.6%) | 66(41.0%) | <0.001 |
