# Supplementary figures and images for: Identification of PANoptosis-Based Prognostic Signature for Predicting Efficacy of Immunotherapy and Chemotherapy in Hepatocellular Carcinoma
Source: Genet Res (Camb). 2023 Jun 5;2023:6879022. doi: 10.1155/2023/6879022 (PMC10260314; doi:10.1155/2023/6879022)

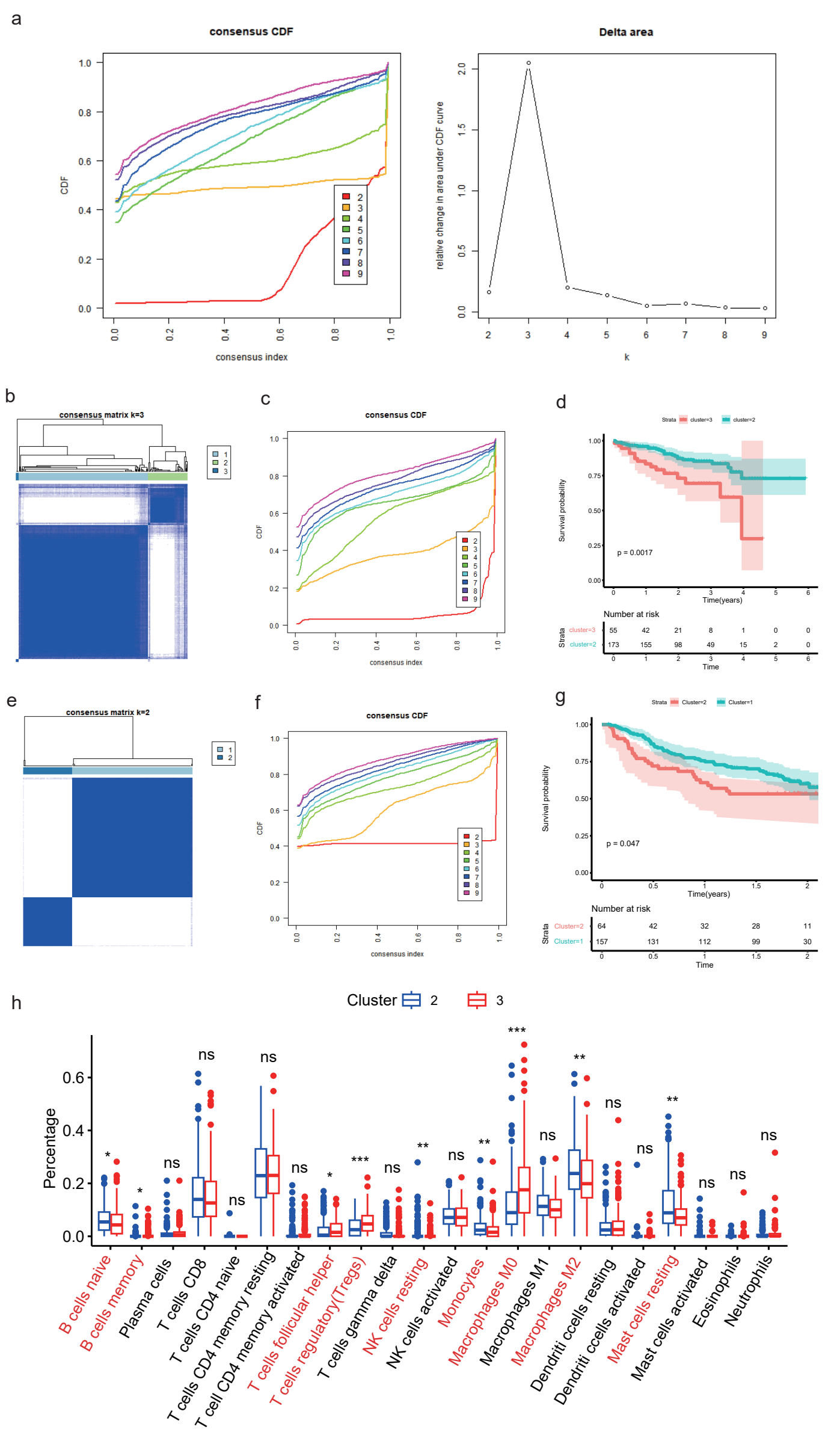

Supplement: Supplementary Materials — Figure S1: Validation of the HCC cluster in validation cohorts. Figure S2: The characteristics of immune infiltration in different risk groups in validation cohorts. Figure S3: Immune checkpoints and an immunotherapeutic response indicator in validation cohorts. Figure S4: Some important indicators in different risk groups. Figure S5: Nomogram model in validation cohorts. Table S1: 26 PANoptosis-related genes; Table S2: The clinical characteristics of HCC patients in different groups. [file 6879022.f1.zip › Supplementary Figure S1.pdf]

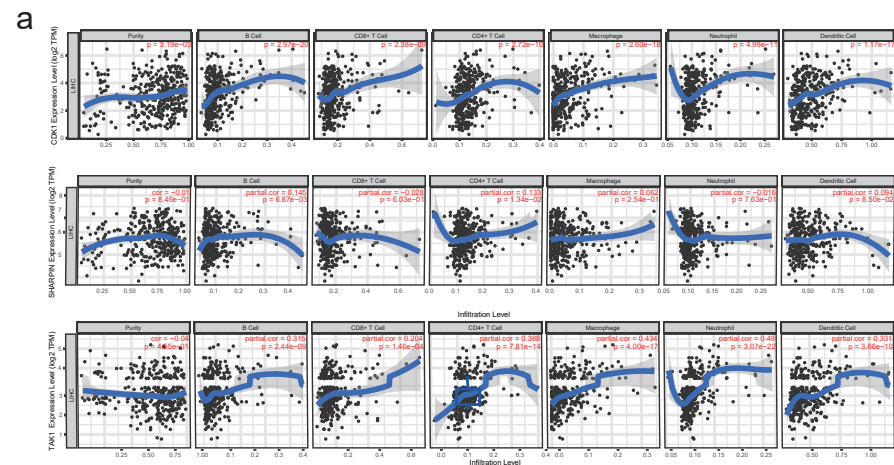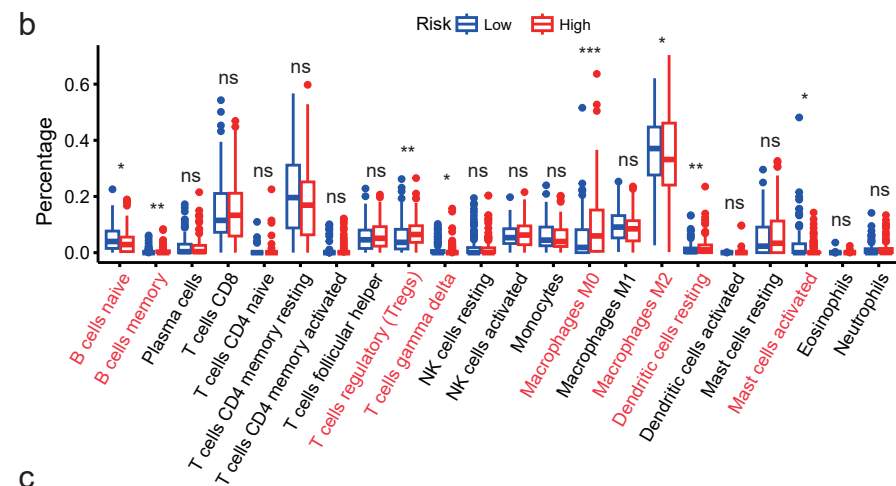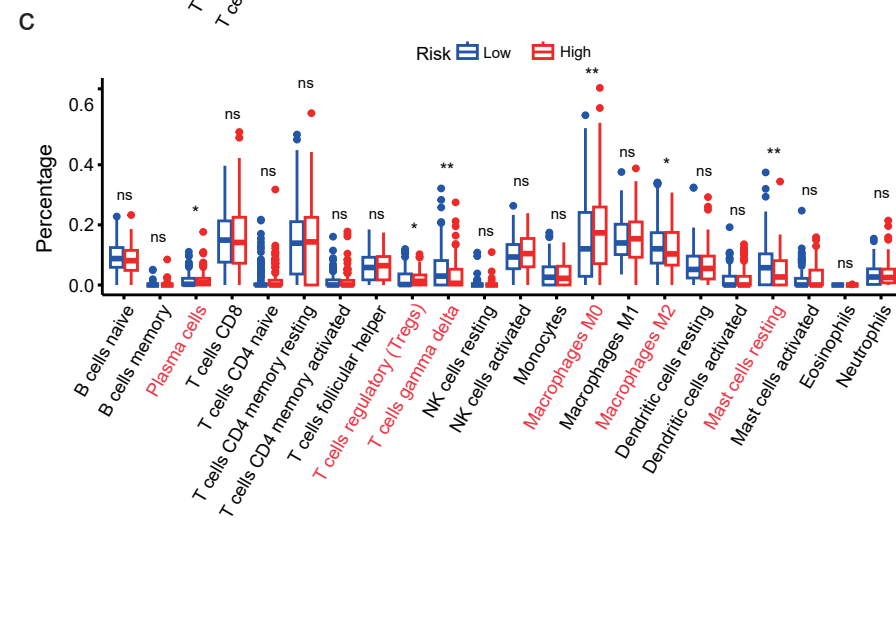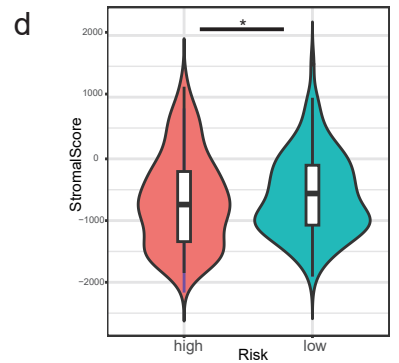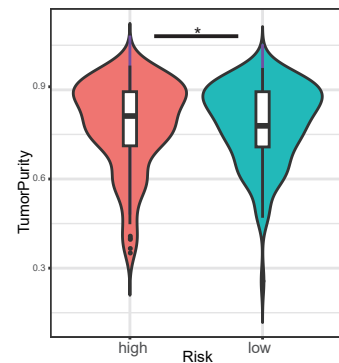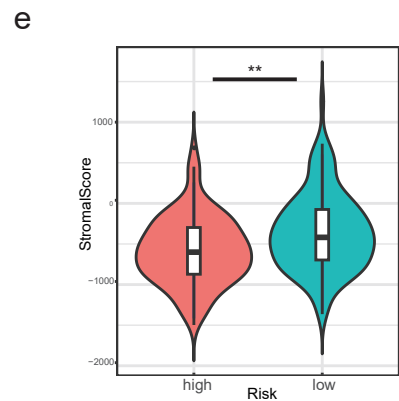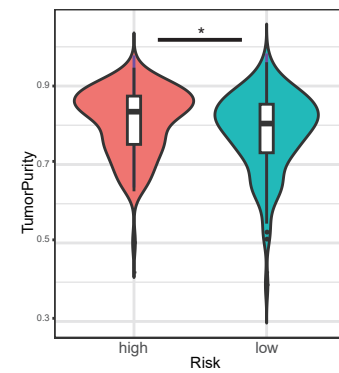

Supplement: Supplementary Materials — Figure S1: Validation of the HCC cluster in validation cohorts. Figure S2: The characteristics of immune infiltration in different risk groups in validation cohorts. Figure S3: Immune checkpoints and an immunotherapeutic response indicator in validation cohorts. Figure S4: Some important indicators in different risk groups. Figure S5: Nomogram model in validation cohorts. Table S1: 26 PANoptosis-related genes; Table S2: The clinical characteristics of HCC patients in different groups. [file 6879022.f1.zip › Supplementary Figure S2.pdf]

a

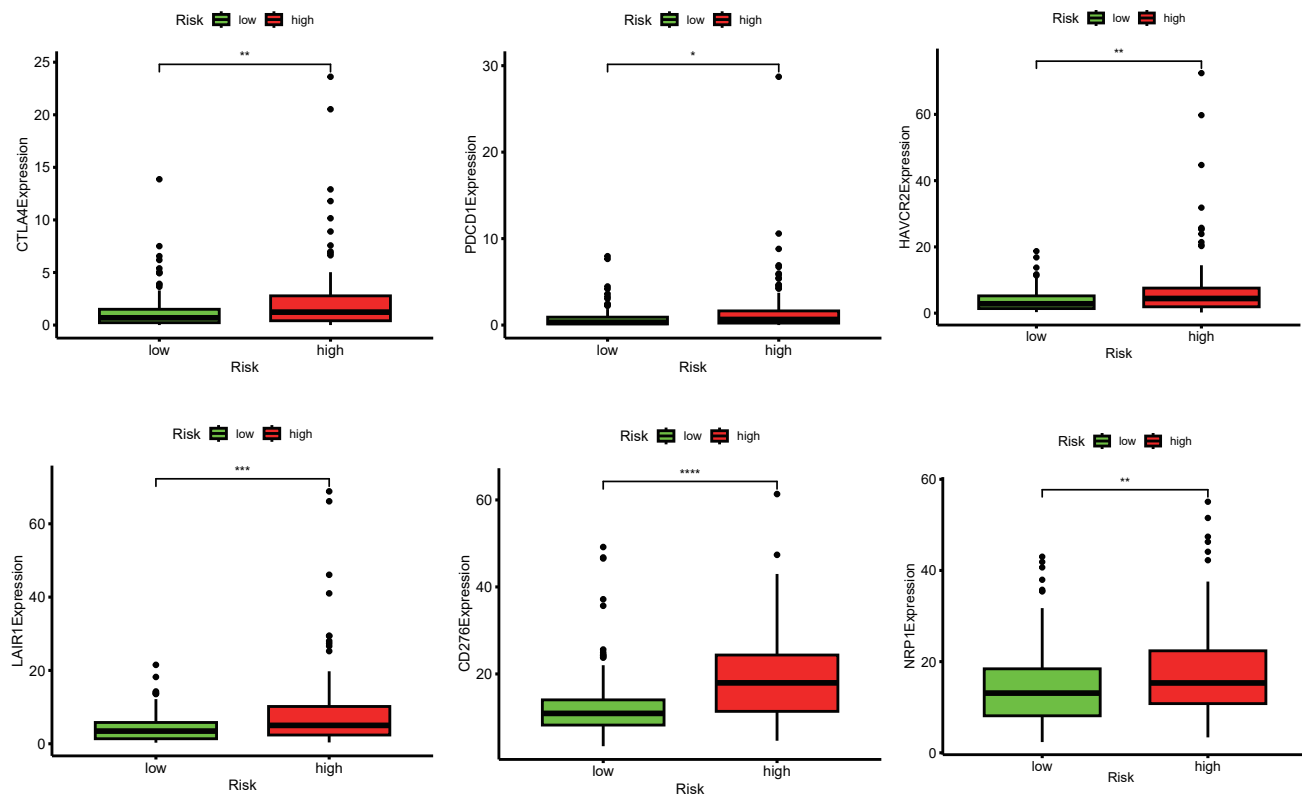

b

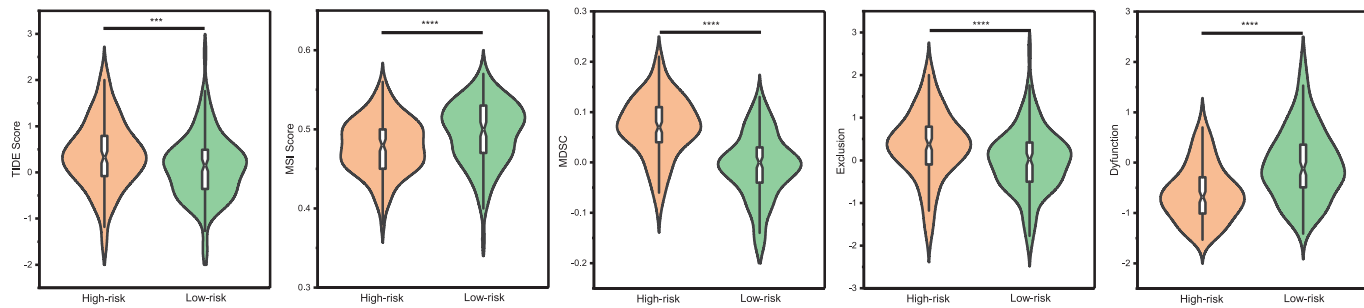

c

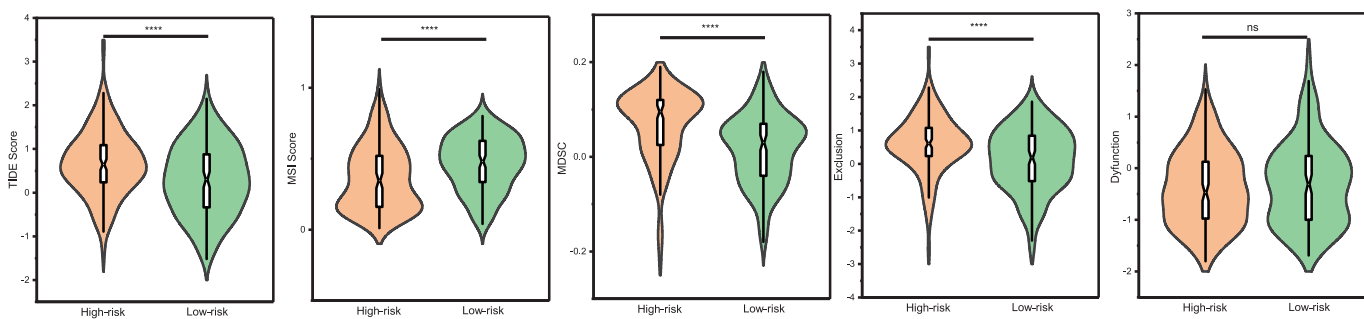

Supplement: Supplementary Materials — Figure S1: Validation of the HCC cluster in validation cohorts. Figure S2: The characteristics of immune infiltration in different risk groups in validation cohorts. Figure S3: Immune checkpoints and an immunotherapeutic response indicator in validation cohorts. Figure S4: Some important indicators in different risk groups. Figure S5: Nomogram model in validation cohorts. Table S1: 26 PANoptosis-related genes; Table S2: The clinical characteristics of HCC patients in different groups. [file 6879022.f1.zip › Supplementary Figure S3.pdf]

**a**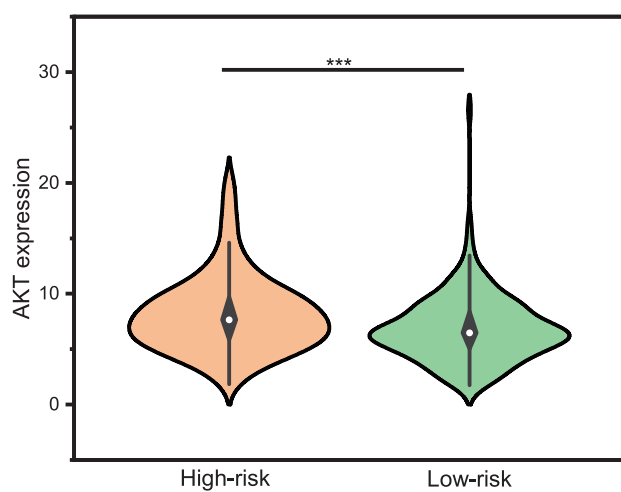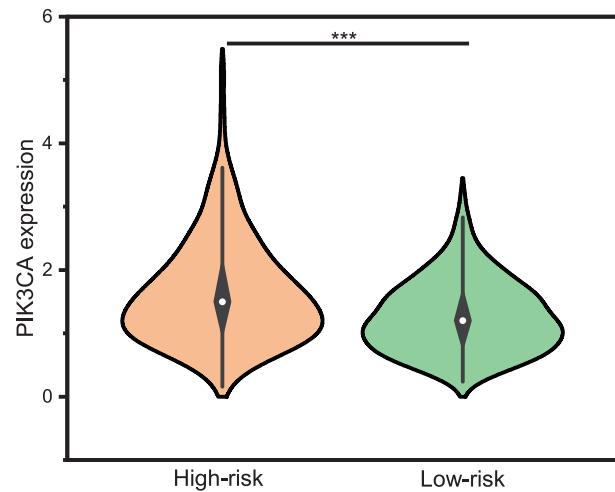**b**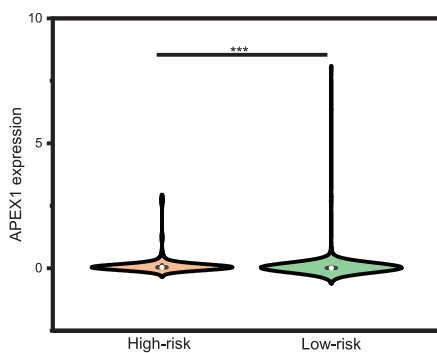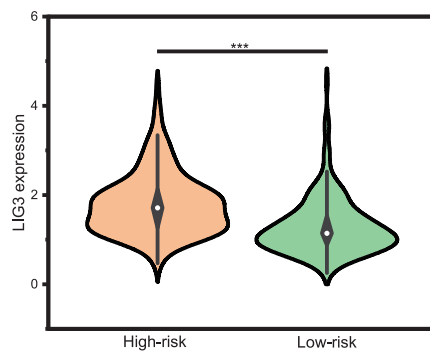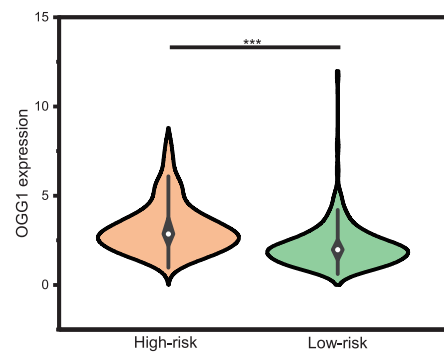**c**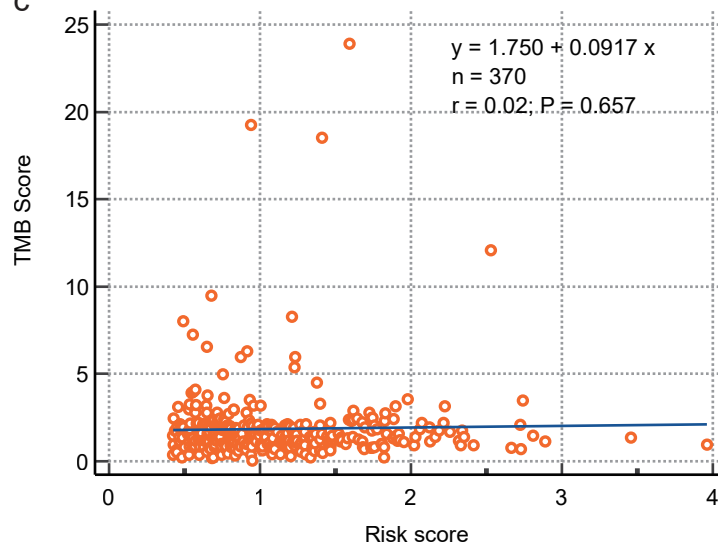

Supplement: Supplementary Materials — Figure S1: Validation of the HCC cluster in validation cohorts. Figure S2: The characteristics of immune infiltration in different risk groups in validation cohorts. Figure S3: Immune checkpoints and an immunotherapeutic response indicator in validation cohorts. Figure S4: Some important indicators in different risk groups. Figure S5: Nomogram model in validation cohorts. Table S1: 26 PANoptosis-related genes; Table S2: The clinical characteristics of HCC patients in different groups. [file 6879022.f1.zip › Supplementary Figure S4.pdf]

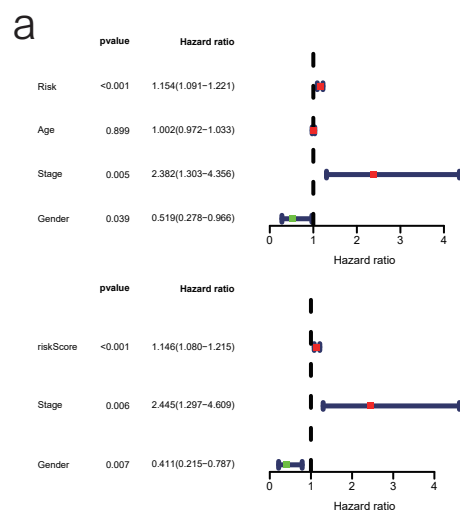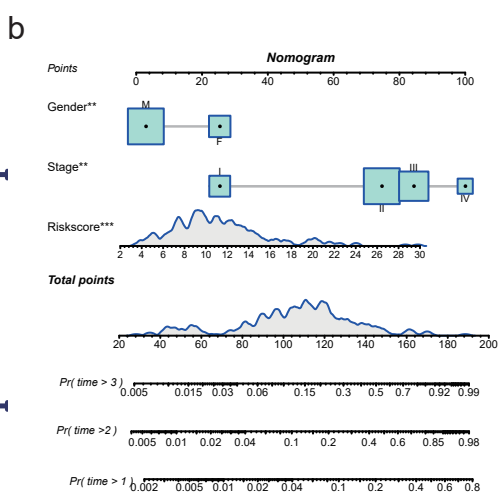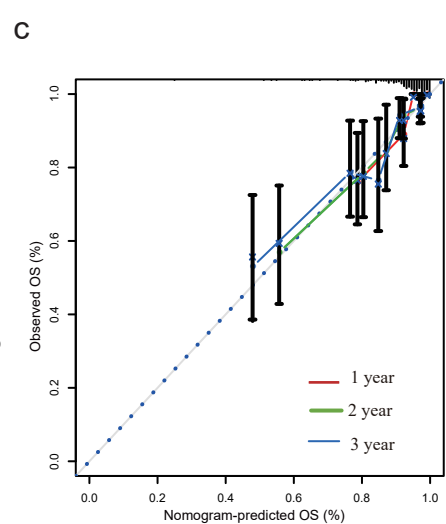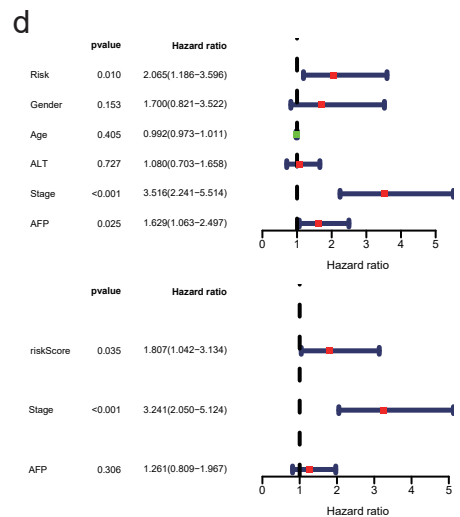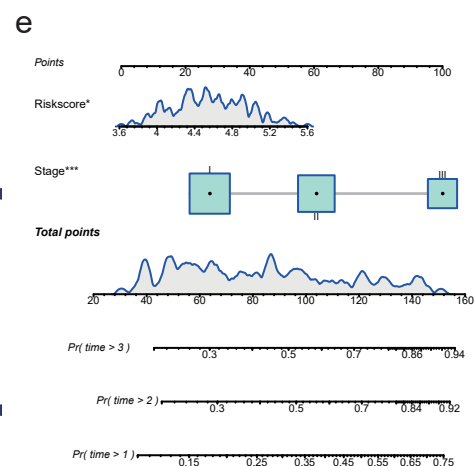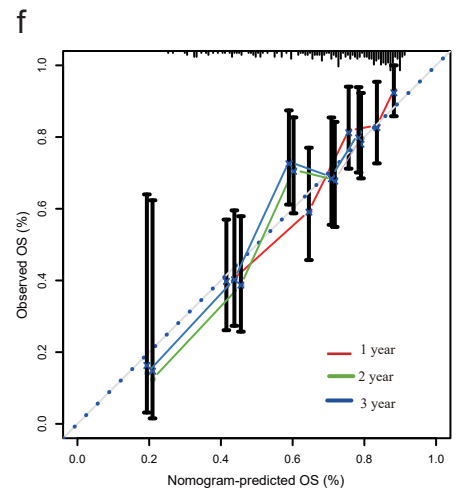

Supplement: Supplementary Materials — Figure S1: Validation of the HCC cluster in validation cohorts. Figure S2: The characteristics of immune infiltration in different risk groups in validation cohorts. Figure S3: Immune checkpoints and an immunotherapeutic response indicator in validation cohorts. Figure S4: Some important indicators in different risk groups. Figure S5: Nomogram model in validation cohorts. Table S1: 26 PANoptosis-related genes; Table S2: The clinical characteristics of HCC patients in different groups. [file 6879022.f1.zip › Supplementary Figure S5.pdf]
